# Investigating the Site‐Specific Impact of Fluorine Substitution on Aromatic Interactions in a Tryptophan Zipper Peptide

**DOI:** 10.1002/chem.202501263

**Published:** 2025-06-29

**Authors:** David Reiter, Ferhat Mutlu, Dominik Ebert, Marceline Noutio, Joachim Heberle, Mario Schubert, Beate Koksch

**Affiliations:** ^1^ Institute of Chemistry and Biochemistry Freie Universität Berlin Arnimallee 20 14195 Berlin Germany; ^2^ Institute of Experimental Physics Freie Universität Berlin Arnimallee 14 14195 Berlin Germany; ^3^ Insitute of Chemistry and Biochemistry Freie Universität Berlin Takustr. 3 14195 Berlin Germany

**Keywords:** fluorine, nmr spectroscopy, peptides, pi interactions, tryptophan

## Abstract

The stability of pairwise tryptophan (Trp) *edge‐to‐face* aromatic interactions has been exploited in the design of small tryptophan zipper (Trpzip) peptides. Herein, we report a systematic study of the regiospecific impact of four constitutional isomers of non‐natural fluoro‐Trp, regarding their incorporation at either *edge*‐ or *face*‐positions. Single fluorine substituents affect the electron density of the indole moiety and introduce a highly electronegative component while the native geometry of Trp is maintained. We employed a library approach based on the sequence of Trpzip2 and assessed peptide structure and stability using CD, FTIR, and NMR spectroscopy. Global hairpin stability was improved or compromised upon site‐specific incorporation of a single monofluoro‐Trp regioisomer. Fluorine substitution revealed key CH/π interactions within the Trp/Trp packing and holds potential for the future optimization of aromatic interactions involving Trp.

## Graphical abstract

Constitutional isomers of fluoro‐tryptophan alter the stability of the β‐hairpin peptide Trpzip2 by tuning key CH/π and NH/π interactions.

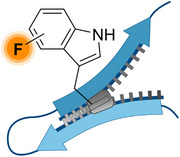

## 1 Introduction

The chemical element fluorine is suspiciously absent in natural organisms: to date only five organofluorine metabolites have been reliably identified.^[^ ^1^ ^]^ Fluorinated amino acids are readily available via organic synthesis and provide biocompatible building blocks for the artificial introduction of fluorine into biological systems.^[^ ^2^ ^]^ Intra‐ and intermolecular geometries are altered by the unique physicochemical properties of the C─F bond,^[^ ^3^ ^]^ presenting a powerful method for modulating stability, folding, and/or binding affinities of peptides and proteins.^[^ ^4^, ^5^, ^6^ ^]^

**Figure 1:**
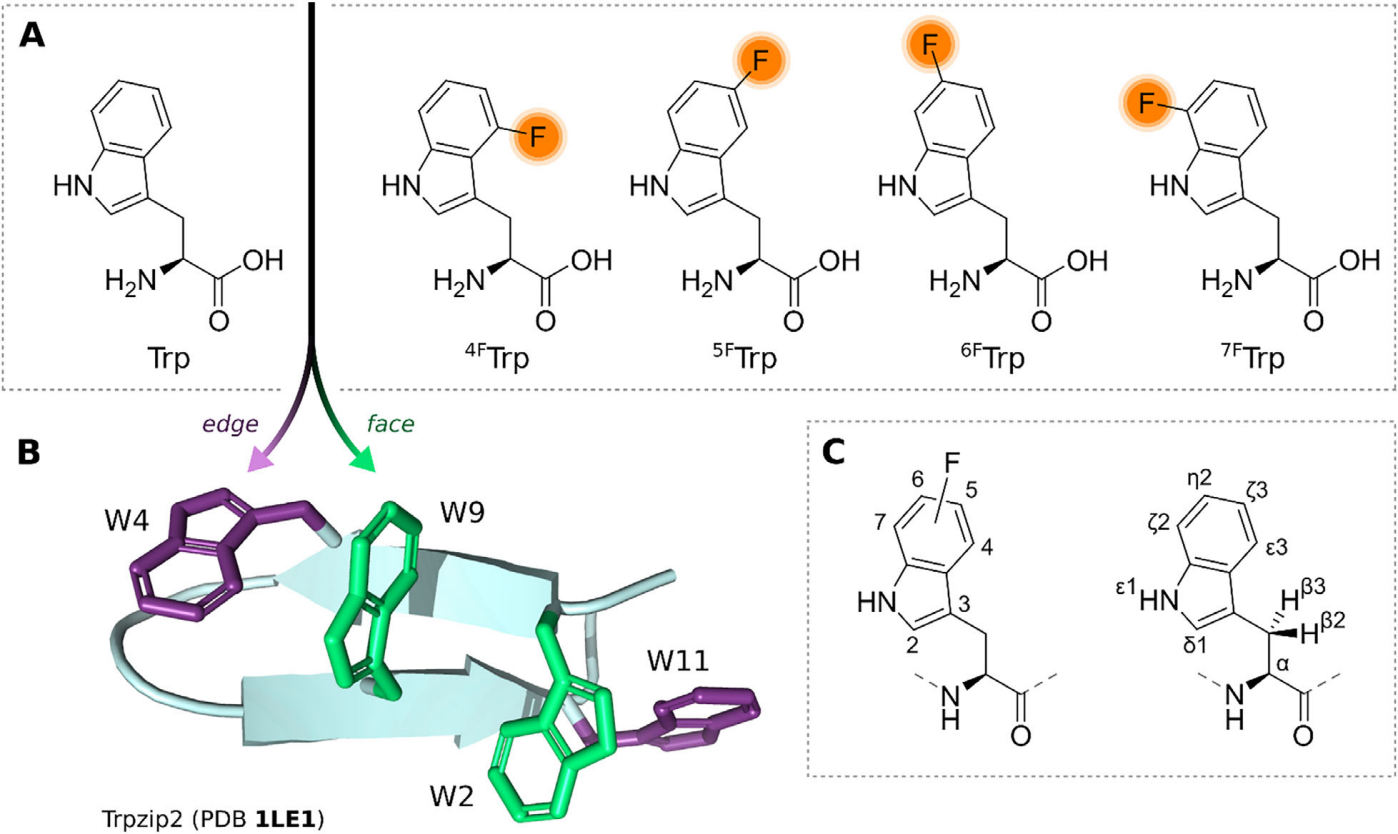
The pairwise interaction of Trp/fluoro‐Trp was investigated in modified variants of the Trpzip2 peptide. A) Chemical structures of native Trp (W), and its 4‐fluoro‐, 5‐fluoro‐, 6‐fluoro‐, and 7‐fluoro‐ analogues. B) β‐hairpin structure of Trpzip2 (PDB code 1LE1).^[^ ^19^ ^]^ *Edge*‐position Trp4, and Trp11 side chains are colored purple, *face*‐position Trp2, and Trp9 side chains are colored green, other side chains are omitted for clarity. C) Indole ring numbering and IUPAC nomenclature of Trp used in this work.

Substitution with tryptophan (Trp) for fluorinated derivatives has been investigated in the context of protein stability,^[^ ^7^ ^]^ protein‐ligand interactions,^[^ ^8^ ^]^ as well as protein‐carbohydrate binding.^[^ ^9^, ^10^, ^11^ ^]^ The monofluorinated 4‐fluoro‐, 5‐fluoro‐, 6‐fluoro‐, and 7‐fluoro‐Trp analogues, shown in Figure 1A, tend to only minimally perturb local structural geometries,^[^ ^7^ ^]^ promoting their use as probes in biomolecular ^19^F‐NMR.^[^ ^12^, ^13^ ^]^ Yet, fluorine affects the electron distribution as well as the dipole moment of tryptophan's aromatic indole moiety,^[^ ^14^, ^15^ ^]^ which may impact CH/π, π‐π, and cation/π involvement. Global effects of fluoro‐Trp substitution seem to be complex and highly dependent on the constitutional isomer. In previous studies on the directed evolution of *E. coli* bacteria towards the uptake of fluorinated amino acid precursors, we observed considerable differences in the organism's acceptance of different fluoroindoles.^[^ ^16^, ^17^ ^]^

The high electron density and dipole moment of the Trp indole moiety allow for both polar interactions, such as hydrogen bonds or π‐interactions, and hydrophobic contacts. Thus, Trp is generally found at positions of structure‐functional importance within the hydrophobic core of proteins or at the interface of the lipid bilayer.^[^ ^18^ ^]^ Trp has become a key building block in the rational design of short yet highly stable *de novo* peptides, such as the tryptophan zippers (Trpzip), a series of β‐hairpins developed by Cochran et al.^[^ ^19^ ^]^ Trpzip and related peptide sequences possess high structural integrity relative to their small size and have become increasingly popular as scaffolds for further decoration. Trpzip scaffolds have been functionalized towards ligand‐ or metal‐binding receptors,^[^ ^20^, ^21^, ^22^, ^23^, ^24^ ^]^ self‐assembling hydrogels,^[^ ^25^, ^26^ ^]^ or mini‐enzymes.^[^ ^27^ ^]^ Trpzip peptides owe their high thermal and chemical stability to the pairwise cross‐strand interactions of two Trp side chains, packed in a T‐shaped *edge‐to‐face* geometry. Out of the original series of tryptophan zipper peptides developed around the turn of the century,^[^ ^19^ ^]^ Tryptophan zipper 2 (Trpzip2, **1**), shown in Figure 1B, in particular, has become a model platform for the study of this Trp‐specific interaction and has been characterized in great detail by the groups of Keiderling and Hauser among others.^[^ ^28^, ^29^, ^30^, ^31^, ^32^ ^]^ These studies determined hydrophobic and aromatic contributions to the total *edge‐to‐face* interaction via a knock‐out approach in which selected Trp residues were substituted for either valine or tyrosine, respectively. More recently, Richaud et al. presented a comprehensive study on the *edge‐to‐face* packing of several non‐natural Trp derivatives incorporated into a custom‐engineered Trpzip hairpin.^[^ ^33^ ^]^ We chose to expand these findings with particular focus on the fluorinated Trp analogues using the well‐defined *edge‐to‐face* interaction of Trpzip2 as a simplified model for the more complex modes of stacking of Trp in larger biological systems. Our aim was to catalogue both energetically favorable, as well as disruptive effects of aryl‐fluorine substituents on the scale of a small peptide model.

In this work we systematically evaluated the interaction of fluorinated derivatives of Trp in the context of the tryptophan zipper motif. We employed a combinatorial approach to investigate the regiospecific effect of fluoro‐Trp substitution at either *edge‐* or *face*‐position by constructing a peptide library based on the sequence of Trpzip2, whose structure, thermodynamics, and kinetics have been investigated in‐depth in previous studies.^[^ ^28^, ^29^, ^30^, ^31^, ^32^ ^]^ Different patterns of fluorine substitution on the indole side chain were explored through site‐specific incorporation of all four monofluoro‐Trp regioisomers, namely 4‐fluoro‐l‐Trp (^4F^Trp), 5‐fluoro‐l‐Trp (^5F^Trp), 6‐fluoro‐l‐Trp (^6F^Trp), and 7‐fluoro‐l‐Trp (^7F^Trp). Stabilizing alkyl‐ and aryl‐XH/π interactions were identified and individually quantified by ^1^H NMR spectroscopy based on their drastic upfield chemical shift deviations as a result of aromatic ring current effects. Our NMR data demonstrate that CH/π components of the *edge‐to‐face* packing can be tuned depending on the position of the fluorine substituent with global impact on hairpin structure and stability. The thermal stability of the Trpzip2 hairpin could be further enhanced through *face*‐position ^4F^Trp9 modification. These findings contribute to the understanding of *edge‐to‐face* stacking on a molecular level and offer insights into the effects of fluorine substitution on CH/π and NH/π interactions in peptides and proteins.

## 2 Results and Discussion

A library of eight peptides was designed and chemically synthesized. Peptide sequences were based on the 12‐amino acids β‐hairpin Trpzip2,^[^ ^19^ ^]^ and contained a single 4‐fluoro (^4F^Trp), 5‐fluoro (^5F^Trp), 6‐fluoro (^6F^Trp), or 7‐fluoro (^7F^Trp) modification of either the Trp4 (**2a**–**2d**) or the Trp9 (**3a**–**3d**) residue, as listed in Table 1. We chose to substitute Trp4/Trp9 in the center of the hairpin and vicinal to the Asn‐Gly loop over the more terminal Trp2/Trp11 pair as we anticipated greater impact on overall peptide folding and stability. Fmoc‐building blocks of all monofluoro‐Trp regioisomers are commercially available and were incorporated by automated microwave‐assisted peptide synthesis, followed by purification of the target peptides on a reversed‐phase high‐performance liquid chromatography (HPLC) system.

**Table 1:** Peptide library and melting temperatures (*T*_m_) determined by CD of 60 µM peptide solutions in 20 mM phosphate buffer (pH 7.0) containing 2 M GuHCl. *T*_m_ values are presented as the means of three independent measurements with their respective standard deviations.

ID	Sequence	*T*_m_ (°C)
**1**	SWTWENGKWT WK–NH_2_	54.1 ± 1.1
**2a**	SWT(^4F^W)ENGKWT WK–NH_2_	44.0 ± 0.9
**2b**	SWT(^5F^W)ENGKWT WK–NH_2_	48.7 ± 0.3
**2c**	SWT(^6F^W)ENGKWT WK–NH_2_	56.9 ± 0.5
**2d**	SWT(^7F^W)ENGKWT WK–NH_2_	56.3 ± 0.9
**3a**	SWTWENGK(^4F^W)T WK–NH_2_	61.5 ± 1.0
**3b**	SWTWENGK(^5F^W)T WK–NH_2_	55.9 ± 0.2
**3c**	SWTWENGK(^6F^W)T WK–NH_2_	53.2 ± 0.6
**3d**	SWTWENGK(^7F^W)T WK–NH_2_	55.0 ± 0.6
**4**	SWT(^4F^W)ENGKWT (^4F^W)K–NH_2_	22.8 ± 1.9
**5**	S(^4F^W)TWENGK(^4F^W)T WK–NH_2_	59.1 ± 0.5

### 2.1 Fluorine Substitution of Arene Donor Protons Reduces Thermal Stability

All fluoro‐Trp modified variants of Trpzip2 showed the expected circular dichroism (CD) spectrum with bands at ∼213 nm, and ∼227 nm, characteristic of the tryptophan zipper fold^[^ ^19^ ^]^ (Figure S1A in the Supporting Information). Exact wavelength values of the maximum and minimum varied slightly given the different photoelectronic properties of the fluoro‐Trp analogues. As previously reported, Trpzip peptides unfold cooperatively with a broad transition from folded to unfolded state.^[^ ^19^, ^30^, ^31^ ^]^ To investigate the thermal stability and determine the melting temperature *T*_m_ of all library peptides, we attempted to record the thermal transition profiles by monitoring the ellipticity at the maximum at ∼227 nm over an increasing temperature from 5 to 95 °C. However, when dissolved in aqueous buffer without additives, peptides did not reach unfolding equilibrium within the specified temperature range. Addition of chaotropic agents such as urea or guanidine hydrochloride (GuHCl) has been shown to lower the melting point of Trpzip peptides by interfering with cross‐strand Trp/Trp interactions.^[^ ^34^ ^]^ We found the addition of 2 M GuHCl to our buffered peptide solutions at pH 7.0 ideal to observe unfolding equilibrium at higher temperatures. Thermal transition profiles (Figure S1B in the Supporting Information) were fit to a two‐state model^[^ ^35^ ^]^ to obtain the melting temperatures given in Table 1.

**Figure 2:**
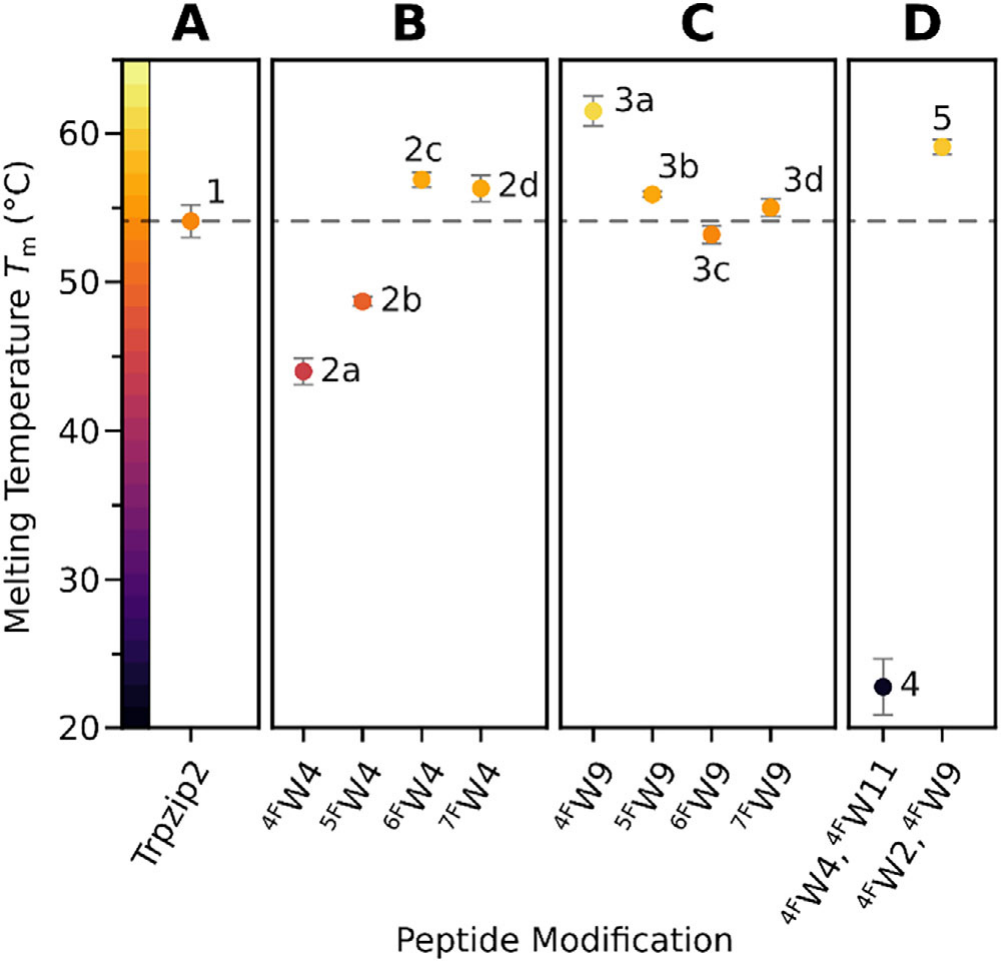
Melting temperatures (*T*_m_) determined via two‐state model fit of peptide thermal transition profiles.^[^ ^35^ ^]^ Melting curves of peptides (60 µM) in phosphate buffer (20 mM, pH 7.0) containing 2 M GuHCl were recorded by monitoring the ellipticity of the maximum at ∼227 nm from 5–95 °C. A) Native peptide **1**. B) *Edge‐*Trp4 substituted peptides (2a–2d) C) *Face*‐Trp9 substituted peptides **3a**–**3d**. **D**. ^4F^Trp double‐substituted peptides **4**, and **5**.

In phosphate buffer (pH 7.0) containing 2 M GuHCl the melting temperature of native Trpzip2 (**1**) was 54.1 °C, as shown in Figure 2A. Substitution for ^4F^Trp4 (**2a**, *T*_m_ = 44.0 °C), or ^5F^Trp4 (**2b**, *T*_m_ = 48.7 °C) reduced the thermal stability drastically, as seen in Figure 2B. Indeed, the substituted aryl‐H^ε3^, and H^ζ3^ protons lie in close proximity to the aromatic plane of the Trp9 benzene ring, as observed in the NMR structure of the native peptide (PDB code 1LE1).^[^ ^19^ ^]^ The observed loss of thermal stability hinted towards the importance of selected individual aryl‐CH/π interactions between Trp4 and Trp9. Conversely, we expected aryl‐fluorination in *ortho*, or *para* position to the CH/π donors to increase thermal stability, which was observed for both the ^6F^Trp4‐, and ^7F^Trp4‐substituted variant (**2c**, *T*_m_ = 56.9 °C; **2d**, *T*_m_ = 56.3 °C). We observed a significantly higher melting temperature for ^4F^Trp9‐modified peptide **3a** (*T*_m_ = 61.5 °C), standing out among the other *face*‐substituted variants, while ^5F^Trp9, ^6F^Trp9, and ^7F^Trp9 had no discernible effect on the global thermal stability, as shown in Figure 2C.

In summary, we observed minor *T*_m_ deviations of ±5 °C against wild type **1** for most fluoro‐Trp substitution patterns. In case of the ^4F^Trp4, and ^5F^Trp4 modification, the observed loss of thermal stability is likely caused by the loss of the respective CH/π interaction and more importantly the expected repulsion between the fluorine substituent and the π‐system in the *edge‐to‐face* packing of ^F^Trp4/Trp9. Surprisingly, the incorporation of ^4^FTrp9 at the *face*‐position increased the global thermal stability.

### 2.2 4‐Fluorotryptophan Alters Thermal Stability and Folding Path

We further investigated the regiospecific impact of ^4F^Trp modification on thermal stability and temperature‐induced unfolding of Trpzip2, as we observed the highest and lowest melting temperatures for ^4F^Trp‐substituted variants **3a**, and **2a**, respectively. Double‐^4F^Trp‐substituted peptides **4** and **5** were subjected to variable temperature CD under previously established conditions in 2 M GuHCl solution. As anticipated based on our results from variant **2a**, substitution of both *edge‐*positions for ^4F^Trp (**4**) resulted in even greater thermal destabilization. As shown in Figure 2D, peptide **4** unfolded at room temperature, which underlines the importance of the two *edge‐*Trp CH^ε3^/π interactions to the global stability of the Trpzip fold. In addition, it is likely that the cation/π interaction between ^4F^Trp11 and the N‐terminal ammonium is compromised, as suggested by previous studies.^[^ ^36^, ^37^, ^38^ ^]^ Substitution of both *face*‐position residues for ^4F^Trp (**5**, *T*_m_ = 59.1 °C), while retaining a higher melting temperature than the native control, however, did not further increase the thermal stability compared to the monosubstituted variant **3a**. This suggests either a highly regiospecific effect of ^4F^Trp9 on the stability of the Trpzip fold or competing stabilizing and destabilizing effects, as we assume that the two fluorine substituents in close proximity might disrupt packing due to steric requirements.

**Figure 3:**
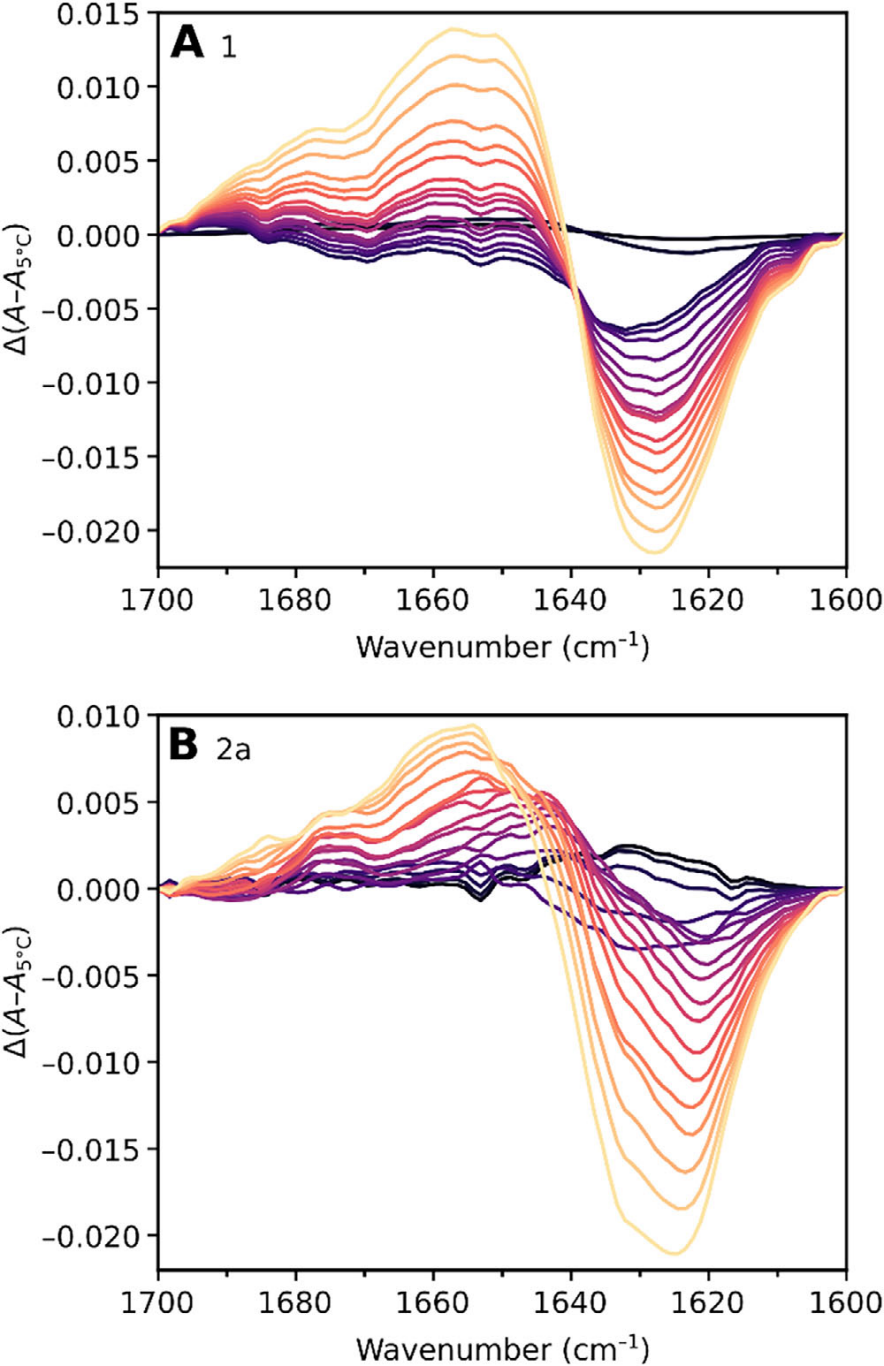
Temperature‐induced FTIR absorption difference spectra in the amide I’ region. Absorption differences (Δ*A*–*A*_5 °C_) correspond to the FTIR spectra recorded in the range of 10–95 °C in 5 °C increments (black to yellow color) subtracted from the FTIR spectrum recorded at 5 °C as a reference. Temperature‐induced changes in the FTIR spectrum of the solvent D_2_O were taken into account. A) Native peptide **1**. The loss of β‐sheet structures at ∼1628 cm^−1^ and the increase of disordered structures at ∼1658 cm^−1^ are indicated by negative and positive bands, respectively. B) ^4F^Trp4 modified peptide **2a**. The loss of β‐sheet structures at ∼1626 cm^−1^ and the increase of disordered structures at ∼1659 cm^−1^ are indicated by negative and positive bands, respectively.

Further insights into the thermal unfolding of **2a** were gained through Fourier Transform Infrared (FTIR) spectroscopy which is superior to CD spectroscopy in discriminating the secondary structure of β‐sheet from random structures.^[^ ^30^, ^31^ ^]^ FTIR absorption spectra of peptide **2a** and native control **1** in D_2_O were recorded from 5 to 95 °C in 5 °C increments. Difference spectra in the amide I’ region, mainly C═O stretching vibration of the peptide bond in D_2_O, were computed by subtracting the FTIR spectrum at 5 °C from those recorded at subsequent temperatures, as shown in Figure 3. The difference spectrum of native Trpzip2 (**1**) displayed a minimum at ∼1628 cm^−1^, and a maximum at ∼1658 cm^−1^ in excellent agreement with previously reported data.^[^ ^39^ ^]^ The frequency of 1628 cm^−1^ of the minimum in the difference spectra indicated a loss in β‐sheet structure and the rise at 1658 cm^−1^ reflected an increase in disordered structure. The presence of an isosbestic point in the difference spectra of peptide **1** indicated direct thermal unfolding of the peptide from the folded to the unfolded state in a cooperative manner. Likewise, the minimum at ∼1626 cm^−1^, and the maximum at ∼1659 cm^−1^ were observable in the difference spectra of peptide **2a**, implying the ^4F^Trp4 modification had no discernible effect on the secondary structure of Trpzip2 in its folded state. However, the absence of an isosbestic point in the difference spectra of **2a** suggested its thermal unfolding proceeded through an alternative intermediate folding state compared to the native control.

### 2.3 ^1^H NMR Reveals Key Interactions within the β‐Hairpin

**Figure 4:**
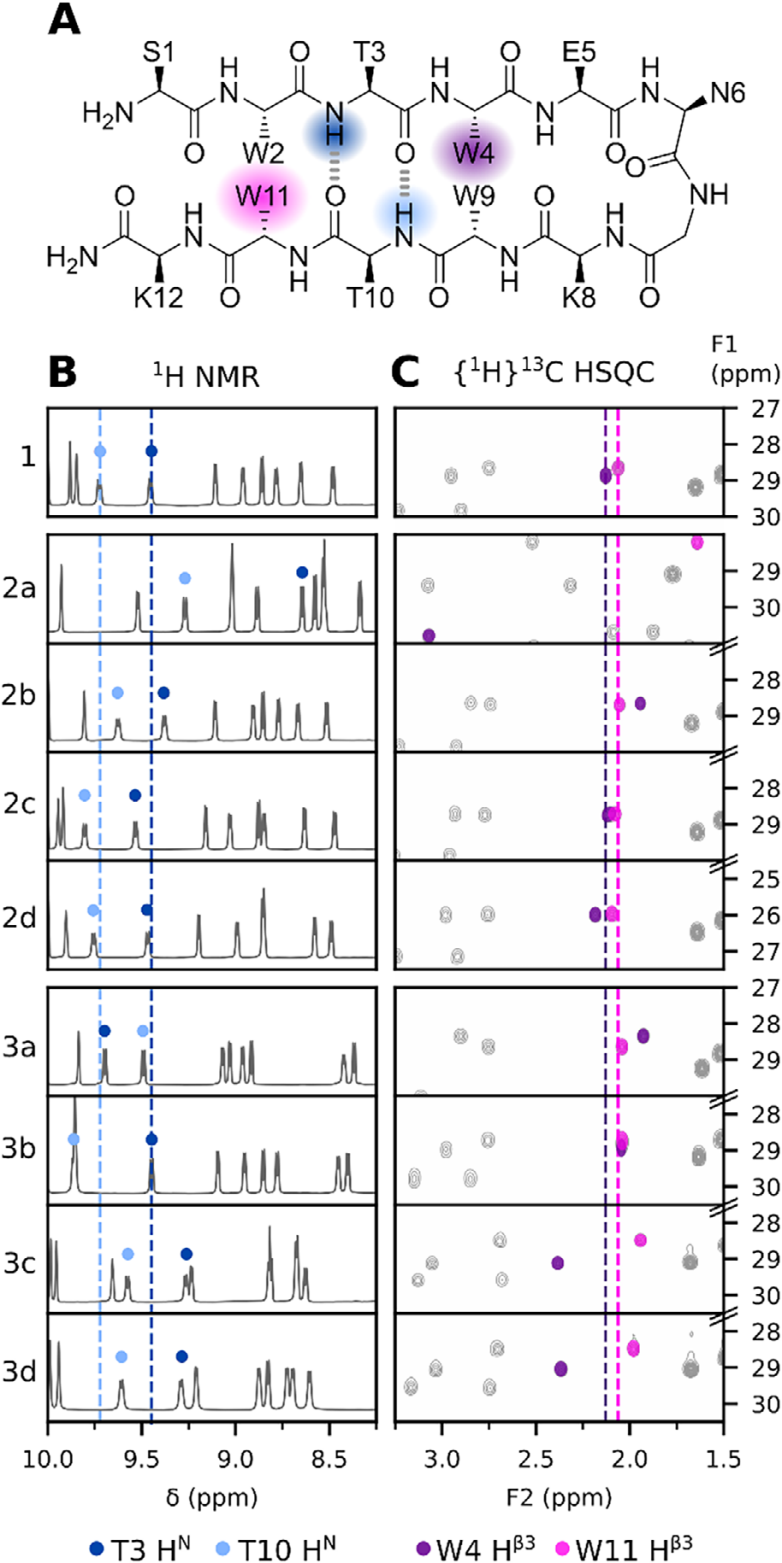
^1^H, and {^1^H}^13^C NMR spectra of native peptide **1**, and fluoro‐Trp monosubstituted variants (2a–3d) A) Schematic representation of the Trpzip2 β‐hairpin. Cross‐strand hydrogen bonds formed by Thr3 HN, and Thr10 HN are colored blue. *Edge‐*position Trp4, and Trp11 are colored purple. B) Thr3 and Thr10 form two cross‐strand hydrogen bonds, contributing to the stability of the Trpzip fold. Downfield Thr3, and Thr10 H^N^ shifts indicate stronger cross‐strand hydrogen bonds. B) Trp4/Trp9, and Trp11/Trp2 are involved in pairwise *edge‐to‐face* interactions. Trp4 Hβ3, and Trp11 Hβ3 are upfield shifted by the ring current effects exerted by the indole moieties of *face*‐Trp9, and ‐Trp2, respectively. Stronger upfield shifted Trp4, and Trp11 H^β3^ shifts indicate closer CH^β3^/π contacts.

To obtain a more detailed picture of dominant interactions within Trpzip2 on the atomic level, we completely assigned ^1^H, ^13^C, ^15^N, and ^19^F chemical shifts of the native peptide **1** and the monosubstituted variants **2a**–**3d** based on 2D NMR experiments (Tables S1–S9 and Figure S2 in the Supporting Information). Key proton signals were identified based on their chemical shift deviation (CSD) against the random coil values reported by Wishart et al.^[^ ^40^ ^]^ Experimentally determined random coil shifts of monofluoro‐Trp regioisomers ^4F^Trp, ^5F^Trp, ^6F^Trp, and ^7F^Trp had not been reported before. We therefore determined the chemical shifts of four synthetic hexapeptides GG(^F^Trp)AGG from 2D NMR spectra recorded under the previously reported conditions^[^ ^40^ ^]^ (Table S10 in the Supporting Information). Unusually large ^1^H CSDs often hint towards hydrogen donor contributions, thus, we compared CSDs across the library peptides to identify potential interactions within Trpzip2. For wild type **1** we calculated pronounced downfield amide H^N^ CSDs for Thr3 (+1.30 ppm), and Thr10 (+1.57 ppm) as a result of cross‐strand hydrogen bonds in the center of the hairpin, shown schematically in Figure 4A. Additionally, we observed large upfield CSDs in *edge*‐position residues Trp4 (H^ε3^ −1.83 ppm, H^β3^ −1.14 ppm), and Trp11 (H^ε3^ −2.30 ppm, H^β3^ −1.21 ppm) for protons in close proximity to the indole moieties of Trp9, and Trp2, respectively. Protons located closely above a π‐ring system are subjected to ring current effects resulting in a drastic upfield shift, correlating to distance and position relative to the center of the aromatic plane.^[^ ^41^, ^42^ ^]^ As such, we argue that the strength of individual XH/π interactions could be estimated based on the donor protons’ upfield CSD. Pronounced upfield CSDs of Gly7 H^N^ (−0.77 ppm), and Lys8 H^N^ (−1.39 ppm) further indicated NH/π contacts between Trp4 and the adjacent turn motif in peptide **1**. Similarly, the CSD of Ser1 H^α^ (−0.91 ppm) suggested a cation/π interaction between the N‐terminal ammonium and Trp11, however, given the rapid hydrogen exchange of cationic species, Ser1 NH_3_^+^ was not observed.

Whereas peptides **2a**–**2d**, and **3a**–**3d** showed qualitatively similar overall ^1^H CSDs to native peptide **1**, we observed a drastically different shift pattern for ^4F^Trp4 substituted **2a** (Figure S3 in the Supporting Information). We therefore decided to first discuss peptides with overall similar ^1^H chemical shifts before interpreting the data of **2a**. Proton signals with large ^1^H chemical shift deviations in wild type **1** served as reporters to compare changes in key interactions across the peptide library. We chose to estimate the strength of the cross‐strand hydrogen bonds based on the amide H^N^ chemical shifts of Thr3, and Thr10, as shown in Figure 4B. Likewise, Trp/Trp contacts were evaluated by comparing {^1^H}^13^C HSQC signals of *edge*‐Trp4 H^β3^, and *edge*‐Trp11 H^β3^, shown in Figure 4C. Hereby we opted for the chemical shift of alkyl‐H^β3^ rather than aryl H^ε3^ to eliminate the potential bias of vicinal effects from fluorine substituents on the indole moiety.

As evident by the investigated reporter shifts, presented in Figure 4, all modified peptides with the exception of **2a** showed a high degree of structural conservation. This was further underlined by our analysis of the NOE cross‐peaks in the two variants with the highest thermal stability (**2c**, **3a**), which were virtually identical to the native control (Table S11 in the Supporting Information). The downfield‐shift of the Thr H^N^ signals in peptides **2c**, **2d**, and **3b** likely correspond to slightly more stable cross‐strand hydrogen bonds, in agreement with the observed increased thermal stability of these peptides. Surprisingly, for the most stable variant **3a**, the Thr H^N^ shifts appeared swapped, implying that Thr3 H^N^ is shifted downfield while Thr10 H^N^ is shifted upfield compared to its native value. Peptides **2b**, **3c**, and **3d** with similar or lower melting temperatures to the wild type **1**, displayed upfield shifted Thr H^N^ signals, suggesting less stable cross‐strand hydrogen bonds. Noticeably, in peptides **3c** and **3d**, the upfield shifts of Trp4 H^β3^ were also less pronounced, which we argue to be caused by a weaker CH^β3^/π interaction in the packing of Trp4 with ^6F^Trp9, or ^7F^Trp9, respectively. Conversely, the ^4F^Trp9‐ (**3a**) and ^5F^Trp9‐substition (**3b**) seemed to tighten the *edge‐to‐face* packing, as evident by the upfield Trp4 H^β3^ shifts as well as the overall increased thermal stability.

Unlike the other peptides **2b**–**3d**, ^4F^Trp4‐substituted peptide **2a** showed drastic chemical shift deviations against the wild type. Amide H^N^ shifts of Thr3 (−0.80 ppm against **1**), and Thr10 (−0.45 ppm against **1**) as well as the downfield shift of ^4F^Trp4 H^β3^ (+0.95 ppm against **1**) indicated the destabilization of the β‐hairpin as the ε3‐fluorine substituent disrupted the interaction of ^4F^Trp4/Trp9. Conversely, the upfield shift of Trp11 H^β3^ (−0.42 ppm against **1**) suggested a tighter *edge‐to‐face* packing of Trp11/Trp2 in **2a**, which could, however, not compensate for the compromised Trp4/Trp9 interaction in the overall hairpin stability as judged by variant **2a**’s melting temperature.

### 2.4 Fluorine Substituents Site‐Specifically Strengthen or Disrupt the *Edge‐to‐Face* Interaction

**Figure 5:**
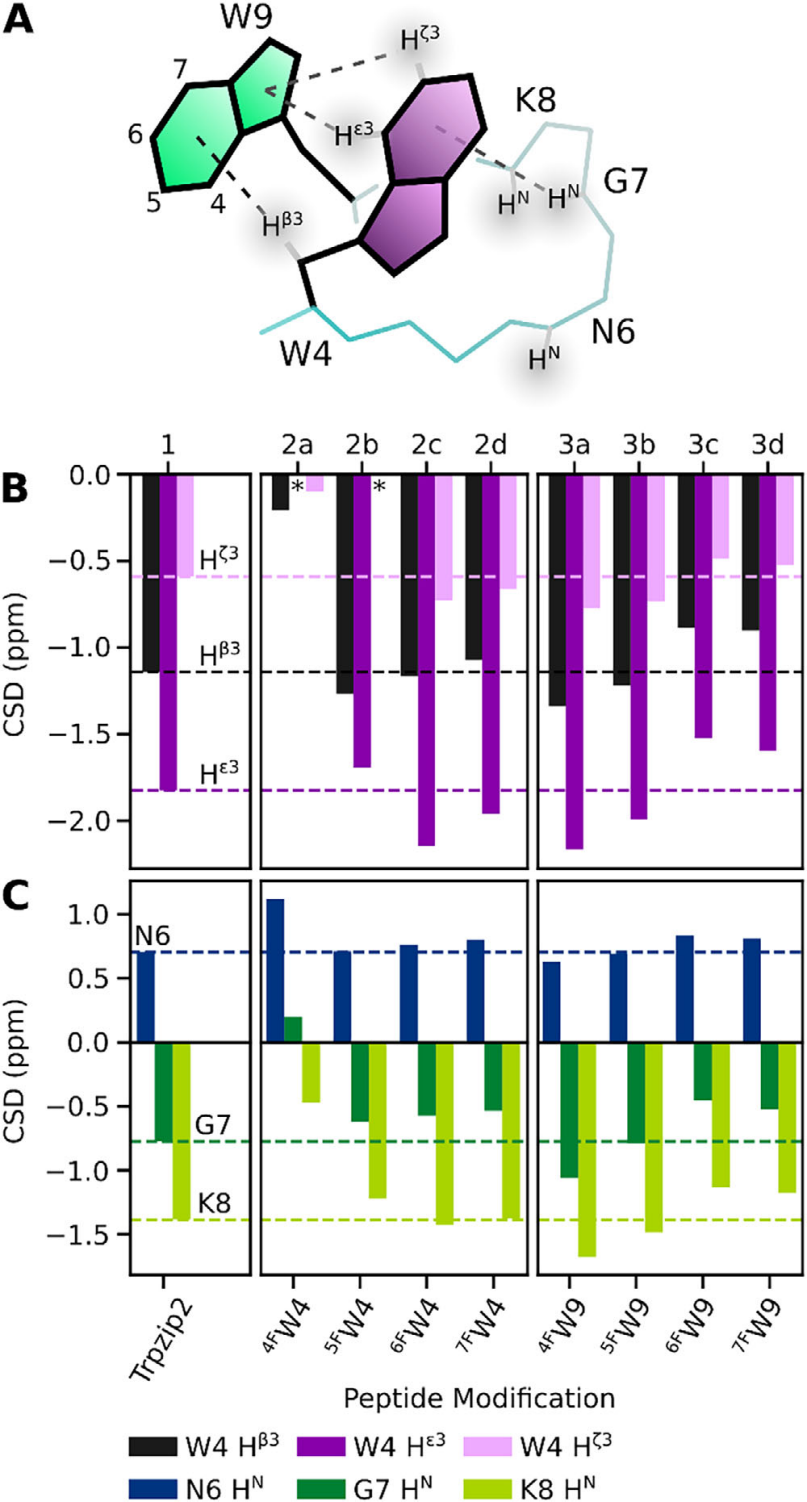
The interactions of Trp4 with Trp9 and the turn motif were assessed from the chemical shift deviations (CSD) of selected protons. CSDs were calculated by subtracting the respective random coil chemical shifts^[^ ^40^ ^]^ from the measured ^1^H chemical shifts. Upfield CSDs are indicative of stronger ring current effects on the proton and therefore closer XH/π contacts. A) Schematic representation of the *edge‐to‐face* packing of Trp4/Trp9, adapted from the solution structure of native Trpzip2 (PDB code 1LE1).^[^ ^19^ ^]^ Dashed lines indicate close CH/π and NH/π contacts with distances ≤4 Å. B) shows CSDs of *edge*‐position (fluoro‐)Trp4 H^β3^, H^ε3^, and H^ζ3^. C) shows CSDs of amide H^N^ protons of Asn6, Gly7, and Lys8 in the turn motif.

^4F^Trp4 substitution in peptide **2a** disrupted the *edge‐to‐face* interaction profoundly. CSDs of remaining Trp4 donors H^β3^ (−0.21 ppm), and H^ζ3^ (−0.10 ppm) were reduced by 80% to native control **1**, indicating a drastic reduction of the otherwise present ring current effect, thus highlighting the disruption of the CH/π interactions. We assume that the fluorine‐ε3 modification not only deletes the central CH^ε3^/pyrrole‐π component, but affects overall *edge‐to‐face* packing though electronegative repulsion of the Trp9 aromatic plane and the ^4F^Trp4 fluorine substituent. Similarly, amide H^N^ protons within the turn motif displayed a much weaker upfield shift, suggesting reorientation of the ^4F^Trp4 side chain and loss of NH/π contributions. Unlike fluorine substitution of H^ε3^ in **2a**, substitution of H^ζ3^ in **2b** had a less drastic impact on the chemical shifts of remaining Trp4 XH/π interactions. The CSD of ^5F^Trp4 H^ε3^ (−1.69 ppm) suggested an increased distance to the Trp9 side chain compared to **1**, while H^β3^ (CSD −1.27 ppm) was further upfield‐shifted. The decrease in strength of turn‐NH/π interactions too were more subtle in peptide **2b** as opposed to **2a**. With electron‐withdrawing fluorine substituents in *meta* (^6F^Trp4, **2c**), and *para* positions (^7F^Trp4, **2d**) to H^ε3^ we anticipated stronger CH/π interactions. Indeed, we calculated larger CSDs for aryl‐H^ε3^ (−2.15 ppm for **2c**, −1.96 ppm for **2d**), and H^ζ3^ (−0.73 ppm for **2c**, −0.66 ppm for **2d**), while CSDs of alkyl‐H^β3^ were comparable to the wild type. CSDs of amide protons in the turn motif were similar to the native values for **2c**, and **2d**. Fluoro‐Trp substitution at the *face*‐position 9 was expected to weaken the *edge‐to‐face* interaction with fluorine withdrawing electron density from the indole‐π system. This was observed for variants containing ^6F^Trp9 (**3c**), and ^7F^Trp9 (**3d**) in which CSDs of the Trp4 donor protons were reduced by 15–20% compared to native control **1**. Additionally, turn‐NH/π components were weakened as judged by the downfield‐shifted Lys8 H^N^, and Gly7 H^N^ signals. ^4F^Trp9 (**3a**), and ^5F^Trp9 modified peptides (**3b**), however, displayed overall enhanced *edge‐to‐face* packing as well as tighter packing of Trp4 and the turn motif. We recorded the most intense upfield CSDs for **3a** with Trp4 H^β3^ (−1.34 ppm), H^ε3^ (−2.17 ppm), and H^ζ3^ (−0.77 ppm) increased by 20–30% compared to native Trpzip2. The overall tightened packing observed in the variants **3a** and **3b**, which is also manifested in their increased thermal stability, is surprising and cannot be explained intuitively based solely on the electronic properties of the ^4F^Trp and ^5F^Trp residues. Possibly, the proximity of the fluorine substituents to the peptide backbone allows for the formation of additionally stabilizing CH···F bonds.

The impact of the fluorine substituents on the unique *edge‐to‐face* packing was investigated in closer detail at the interaction of Trp4/Trp9, shown schematically in Figure 5A. Trp4‐CH/π contacts were monitored based on the ^1^H chemical shift deviation (CSD) of selected donor hydrogens against the random coil values,^[^ ^40^ ^]^ as shown in Figure 5B, assuming stronger interactions with increasingly upfield‐shifted CSDs.^[^ ^41^, ^42^ ^]^ In addition, we evaluated the impact on the adjacent turn motif by comparing CSD of Asn6, Gly7, and Lys8 amide H^N^ protons shown in Figure 5C. For native peptide **1** we calculated distinct upfield CSDs for Trp4 H^ε3^ (−1.83 ppm), and H^β3^ (−1.14 ppm), indicative of their close proximity to the Trp9 indole moiety. H^ζ3^ (CSD −0.59 ppm) too was observed to contribute to the *edge‐to‐face* packing in Trpzip2. Moreover, we found Trp4 to engage with the hairpin turn via stabilizing NH/π interactions with upfield CSDs of Lys8 H^N^ (−1.39 ppm), and Gly7 H^N^ (−0.77 ppm). Our findings align with the recently reported results by Richaud et al. who characterized the Trp/Trp *edge‐to‐face* stacking as an interplay of both alkyl‐, and aryl‐CH/π and NH/π interactions, with the *edge*‐Trp acting as both CH/π donor as well as NH/π acceptor.^[^ ^33^ ^]^

Conclusively, we observed drastic decreases in the strength of both aryl‐, and alkyl‐CH/π, as well as NH/π interactions upon fluorine‐ε3 substitution (^4F^Trp4) on the *edge*‐position, highlighting the importance of the CH^ε3^ donor. The incorporation of fluorine‐ε3 into the core of the Trp4/Trp9 *edge‐to‐face* packing seems to cause the repulsion of both aromatic side chains whereas fluorine‐ζ3 *edge*‐substitution (^5F^Trp4) has no severe structural impact. CSDs of other fluoro‐Trp modified peptides deviated only slightly from the native values, with the most thermally stable ^4F^Trp9‐substituted variant **3a** displaying the largest upfield CSDs of CH/π, and NH/π donor protons overall.

## 3 Conclusion

We present a systematic evaluation of the impact of fluoro‐Trp substitution on stability and fold of a Trpzip motif. All four benzene‐monosubstituted regioisomers of fluoro‐Trp (^4F^Trp, ^5F^Trp, ^6F^Trp, and ^7F^Trp) were incorporated site‐specifically via solid‐phase synthesis into the sequence of Trpzip2, a well‐established model for the study of Trp‐specific stacking interactions. Fluoro‐Trp analogues were paired with native Trp at either *edge‐* or *face‐*positions to assess the individual contribution of each regioisomer on peptide stability and secondary structure. Peptides were characterized on the atomic level by assigning all ^1^H, ^13^C, ^15^N, and ^19^F NMR chemical shifts and comparing the chemical shift deviations against the random coil chemical shifts, which were determined experimentally for the first time for all four monofluoro‐Trp derivatives. Fluorine substitution confirmed the importance of aryl‐CH^ε3^/pyrrole‐π and aryl‐CH^ζ3^/pyrrole‐π interactions on the overall *edge‐to‐face* packing. The strength of individual CH/π, and NH/π interactions varied depending on the position of the fluorine substituent on the indole moiety. Within the scope of our peptide library, we found fluorine‐ε3 substitution on *face*‐Trp9 to have the greatest stabilizing effect overall, raising the peptide melting temperature (*T*_m_) by 7 °C compared to Trpzip2 while conserving the native structure. Incorporation of a single fluorine substituent at *edge‐* ^5F^Trp4 reduced the melting temperature by 5 °C, substitution with *edge*‐^4F^Trp4 by 10 °C. Disrupting both *edge‐to‐*face interactions by incorporating ^4F^Trp4 and ^4F^Trp11 at both edge‐positions resulted in unfolding of the peptide at room temperature. These thermal melts provide an initial guide to assess the relative stability of aromatic fluoro‐Trp interactions, which should be further specified by determining the individual energetic contributions in future studies. The incorporation of a single fluorine substituent into Trpzip2 resulted in overall minor structural perturbations as judged by the ^1^H chemical shift deviations, with the exception of the *edge*‐position ^4F^Trp4 modification, which caused global destabilization of the β‐hairpin. FTIR spectral traits contribute insights into the individual thermal stability and folding path of this ^4F^Trp4‐containing variant. Its thermal unfolding pathway deviates from the native control and involves an alternative folding intermediate. Ultimately, this work increases our understanding of regiospecific effects of aryl‐fluorine substituents in the context of Trp/Trp interactions. Our findings support more precise predictions of stabilizing or disruptive effects of the different fluoro‐Trp regioisomers in peptides and proteins with potential benefits to the development of minimally‐invasive probes for analytical applications, such as ^19^F‐NMR spectroscopy.

## Supporting Information

The authors have cited additional references within the Supporting Information.^[^ ^43^, ^44^, ^45^, ^46^, ^47^ ^]^

## Conflict of Interests

The authors declare no conflict of interest.

## Supporting information



Supporting Information

## Data Availability

The data that support the findings of this study are openly available in Biological Magnetic Resonance Data Bank at https://www.bmrb.io, reference numbers 52965, 52986, 52987, 52988, 52989, 52990, 52991, 52992, 52993, 52994, 52995, 52996, and 52997.
