# Supplementary material for: Investigating the Site‐Specific Impact of Fluorine Substitution on Aromatic Interactions in a Tryptophan Zipper Peptide
Source: Chemistry. 2025 Jun 29;31(40):e202501263. doi: 10.1002/chem.202501263 (PMC12271999; doi:10.1002/chem.202501263)
Supplement: Supplementary file 1 — Supporting Information [file CHEM-31-e202501263-s001.pdf]

Supporting Information

Investigating the Site-Specific Impact of Fluorine Substitution on Aromatic Interactions in a Tryptophan Zipper Peptide

David Reiter, Ferhat Mutlu, Dominik Ebert, Marceline Noutio, Joachim Heberle, Mario Schubert, and Beate Koksch

Contents

1 Supplementary Figures **2**

2 Supplementary Tables **19**

3 Experimental Procedures **31**
3.1 Materials 31
3.2 Software 31
3.3 Solid-Phase Peptide Synthesis 31
3.4 High-Performance Liquid Chromatography (HPLC) 31
3.5 Mass Spectrometry 32
3.6 UV Spectroscopy 32
3.7 Peptide Stock Solutions 33
3.8 Circular Dichroism (CD) Spectroscopy 33
3.9 Fourier Transform Infrared (FTIR) Spectroscopy 34
3.10 Nuclear Magnetic Resonance (NMR) Spectroscopy 34

4 Abbreviations **35**

1 Supplementary Figures

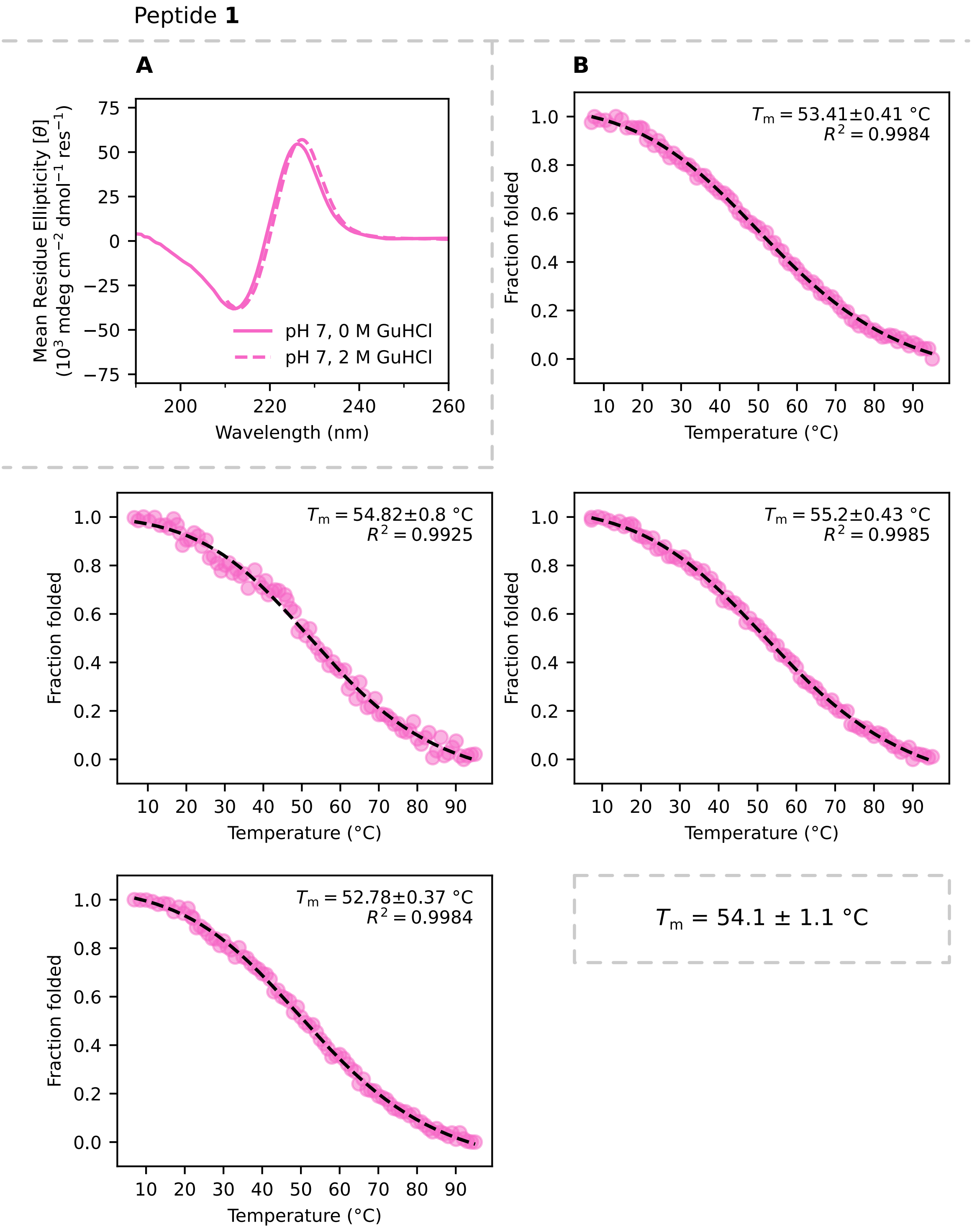

Figure S1. Circular dichroism (CD) spectroscopy of Trpzip2 variants **1**–**5**. **A.** CD spectrum recorded at 20 °C in 20 mM phosphate buffer (pH 7.0) containing 60 µM peptide and 0 M or 2 M GuHCl. **B.** Thermal denaturation profiles recorded in 20 mM phosphate buffer (pH 7.0) containing 60 µM peptide with 2 M GuHCl by monitoring the ellipticity of the maximum at 227 nm from 5–95 °C. Thermal denaturation profiles were fit to a two-state model[1] and the melting temperatures (T_m) from the individual fits were averaged.

Peptide **2a**

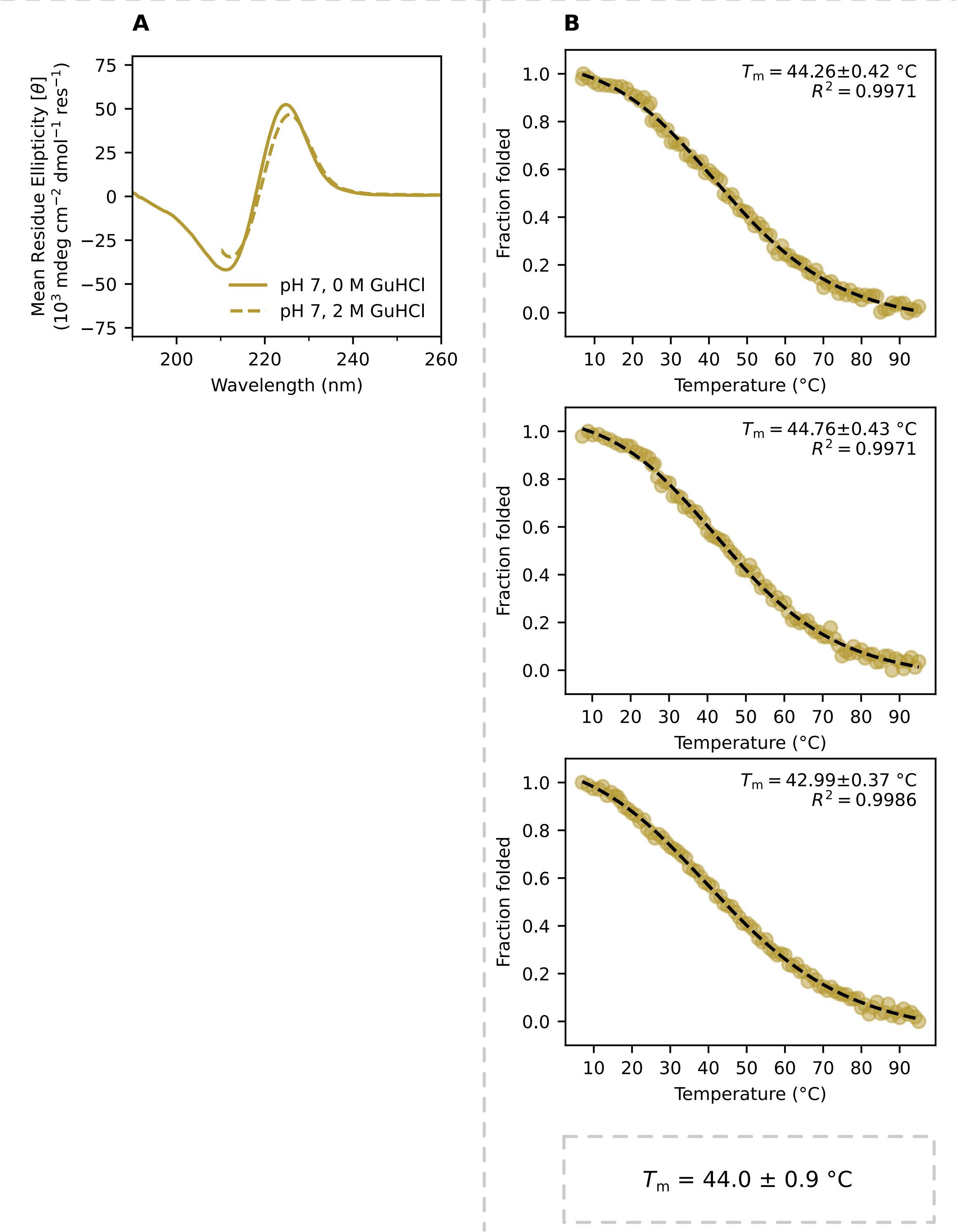

Figure S1 (continued). See complete caption on page 2.

Peptide **2b**

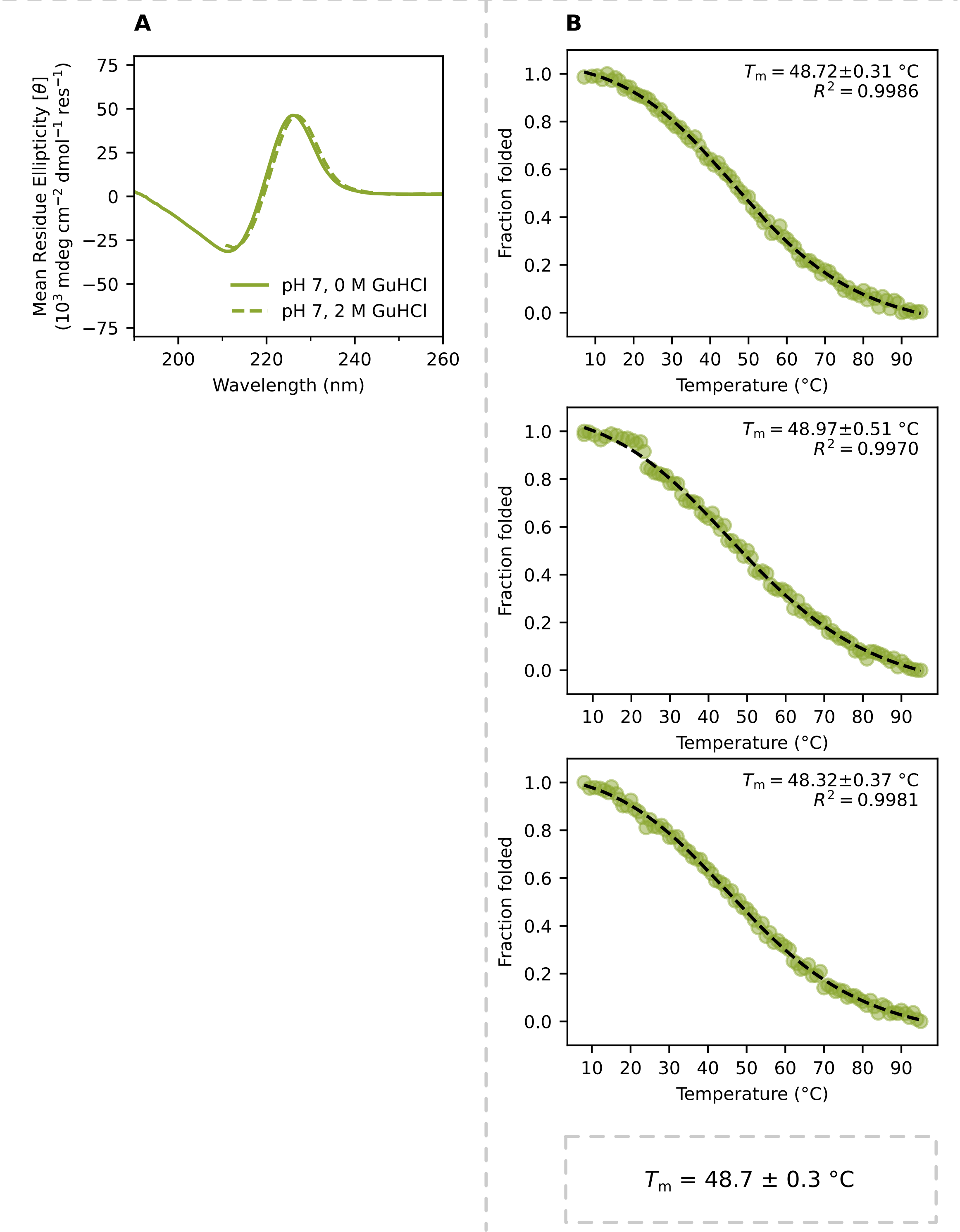

Figure S1 (continued). See complete caption on page 2.

Peptide **2c**

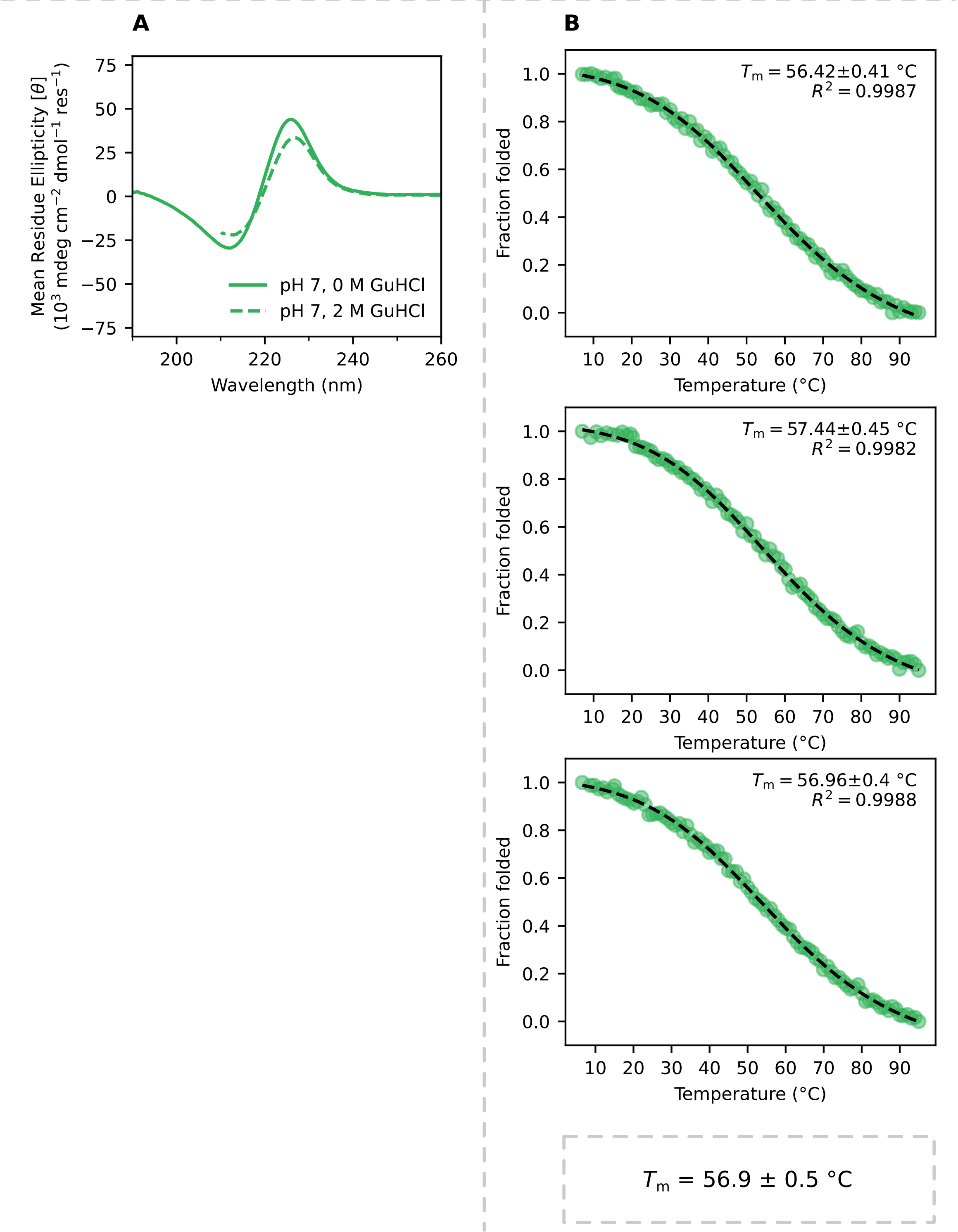

Figure S1 (continued). See complete caption on page 2.

Peptide **2d**

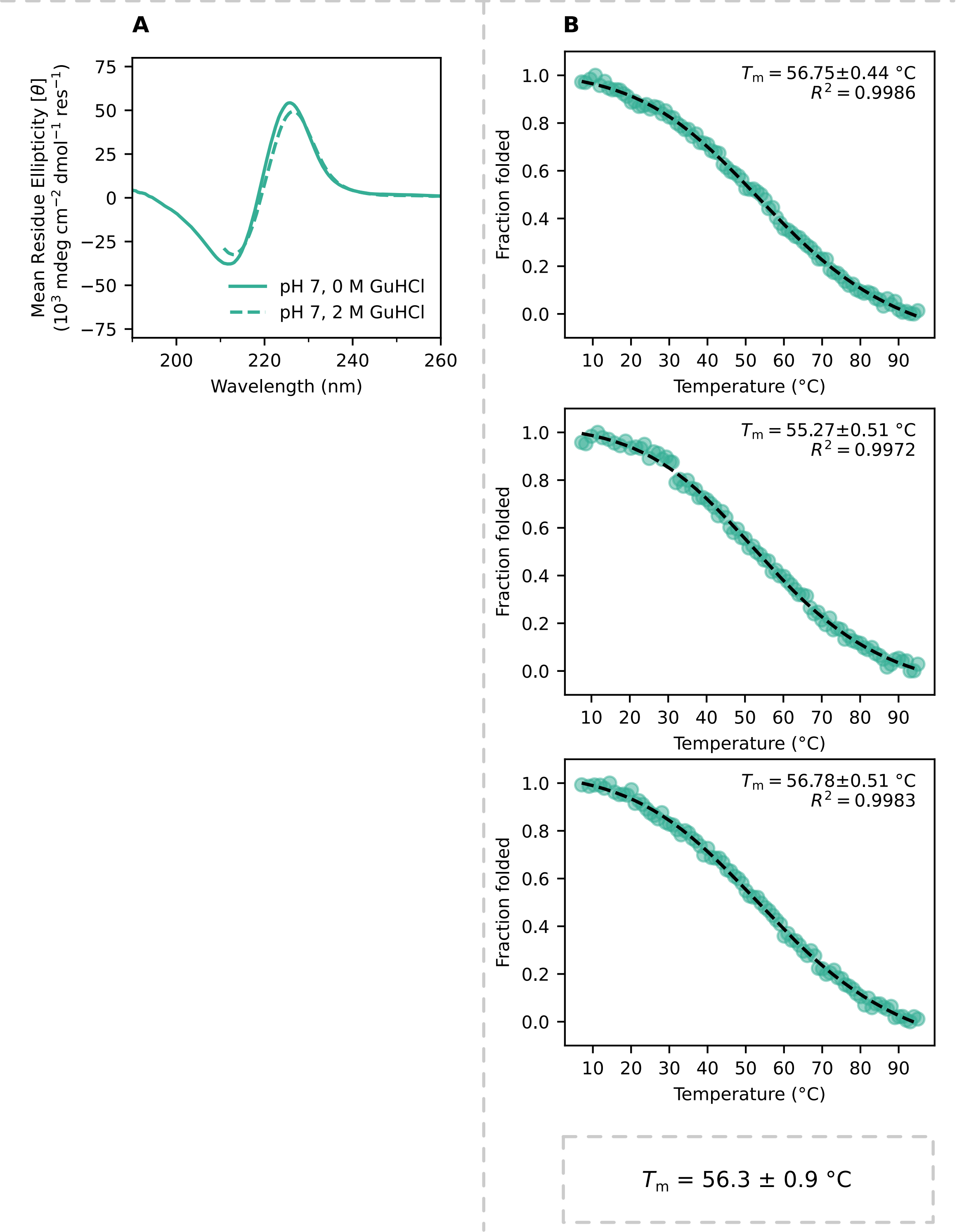

Figure S1 (continued). See complete caption on page 2.

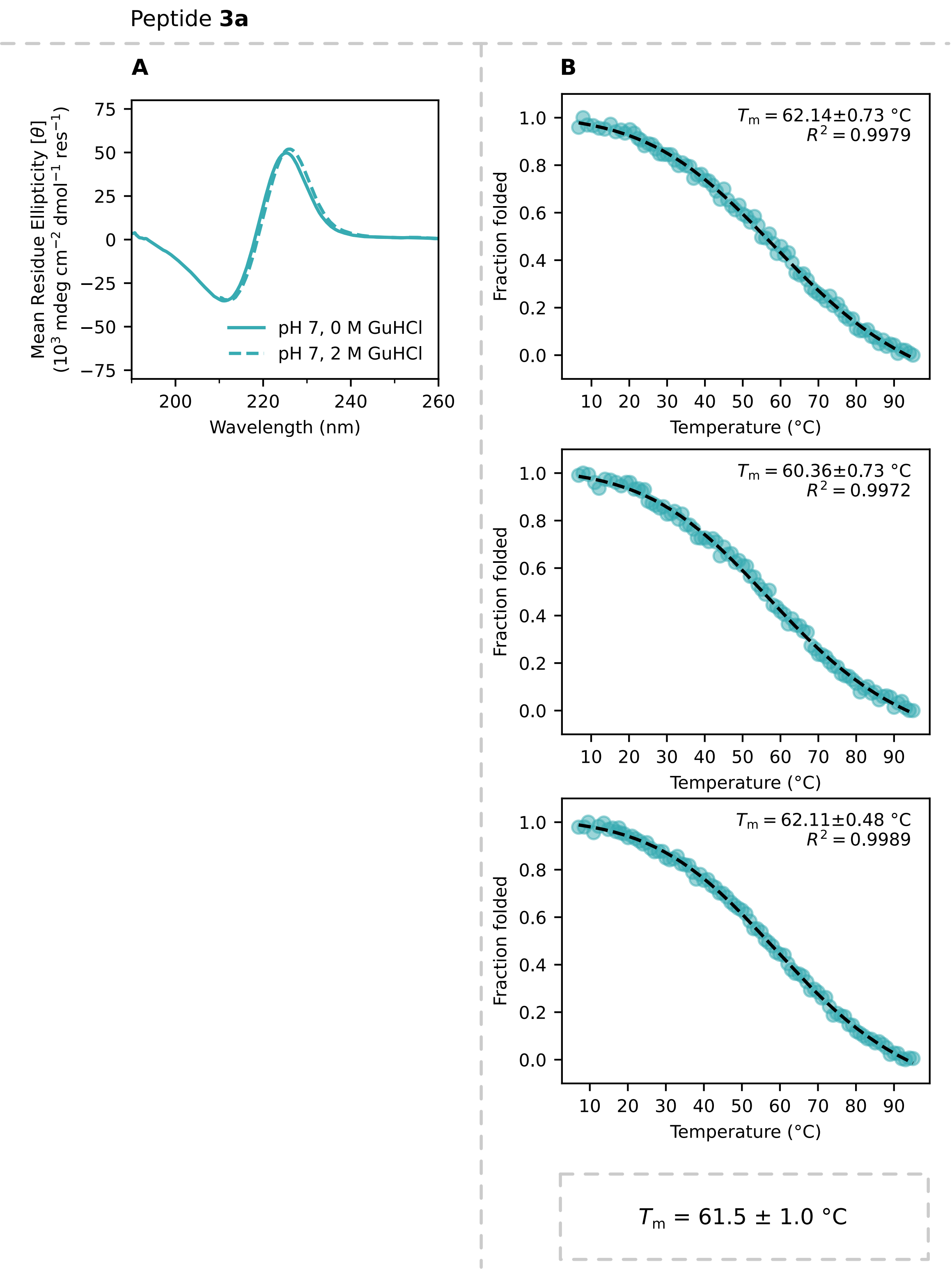

Figure S1 (continued). See complete caption on page 2.

Peptide **3b**

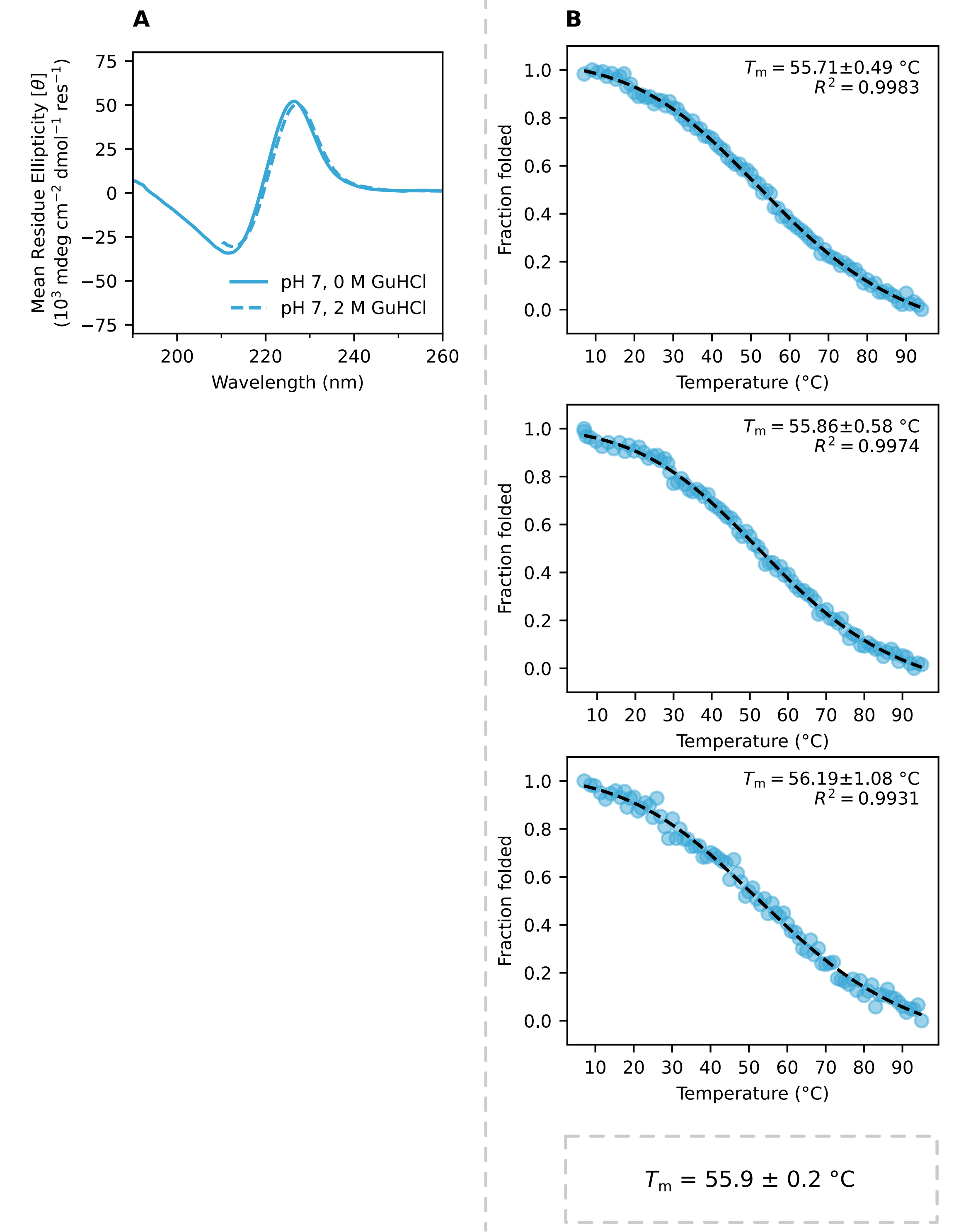

Figure S1 (continued). See complete caption on page 2.

Peptide **3c**

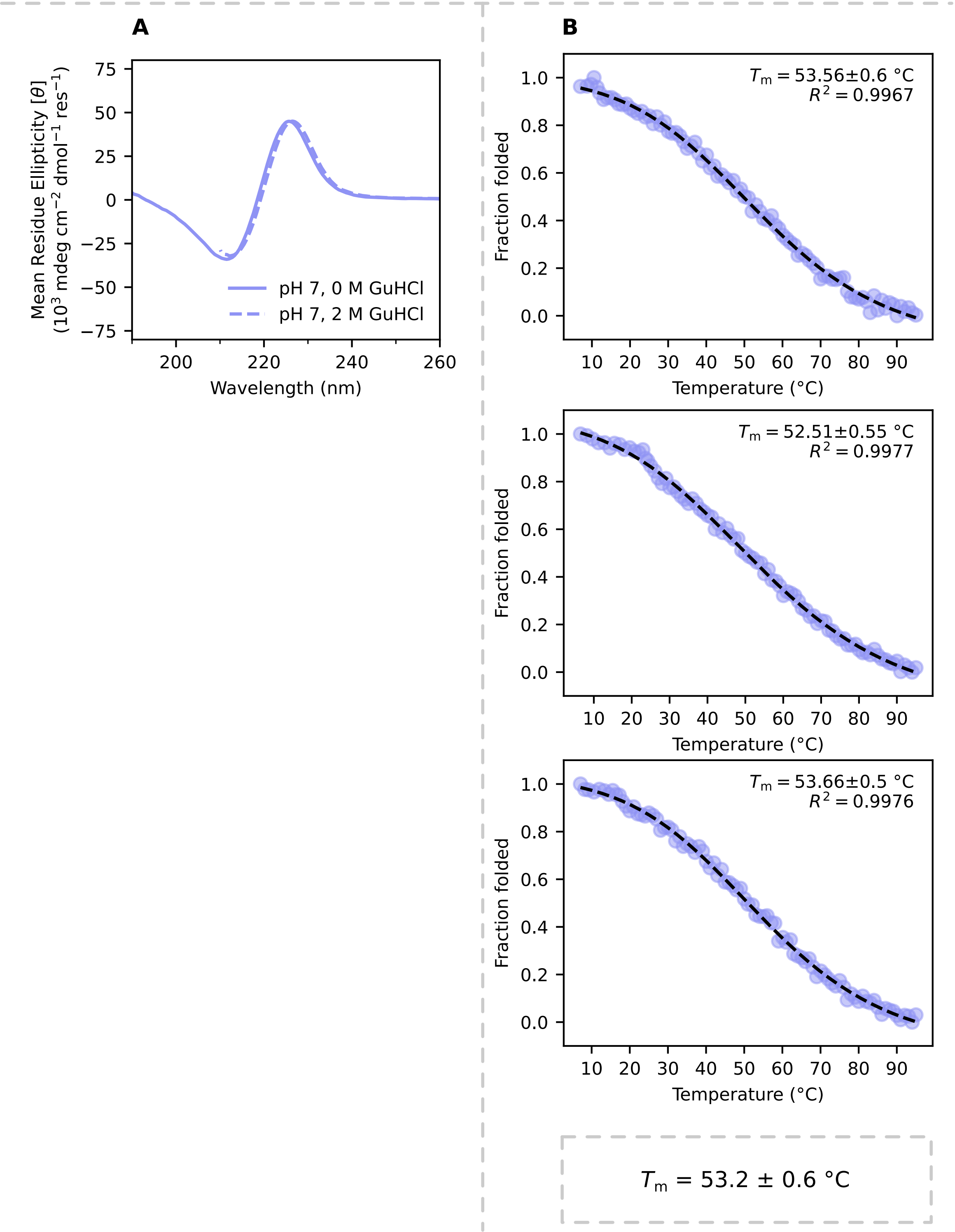

Figure S1 (continued). See complete caption on page 2.

Peptide **3d**

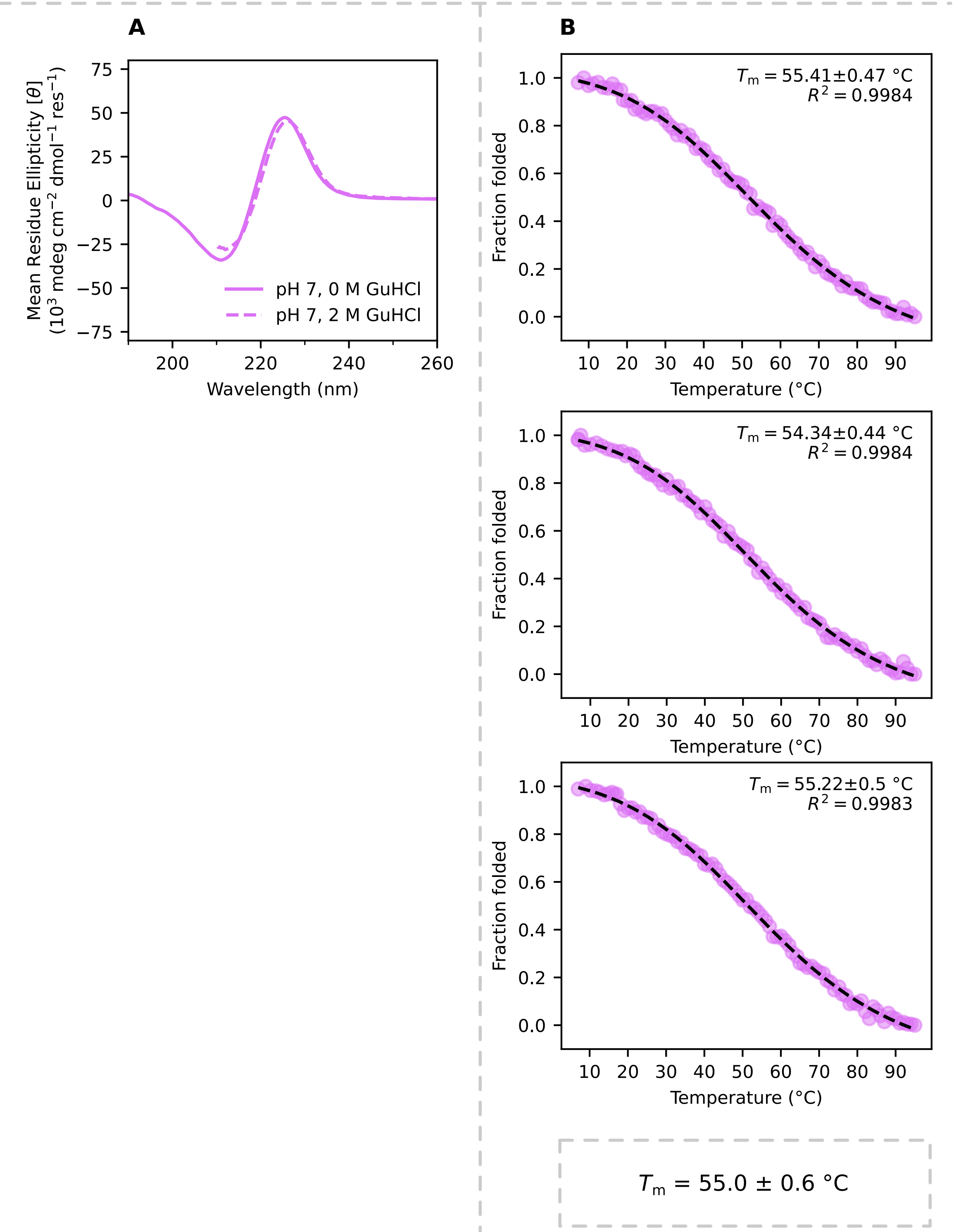

Figure S1 (continued). See complete caption on page 2.

Peptide **4**

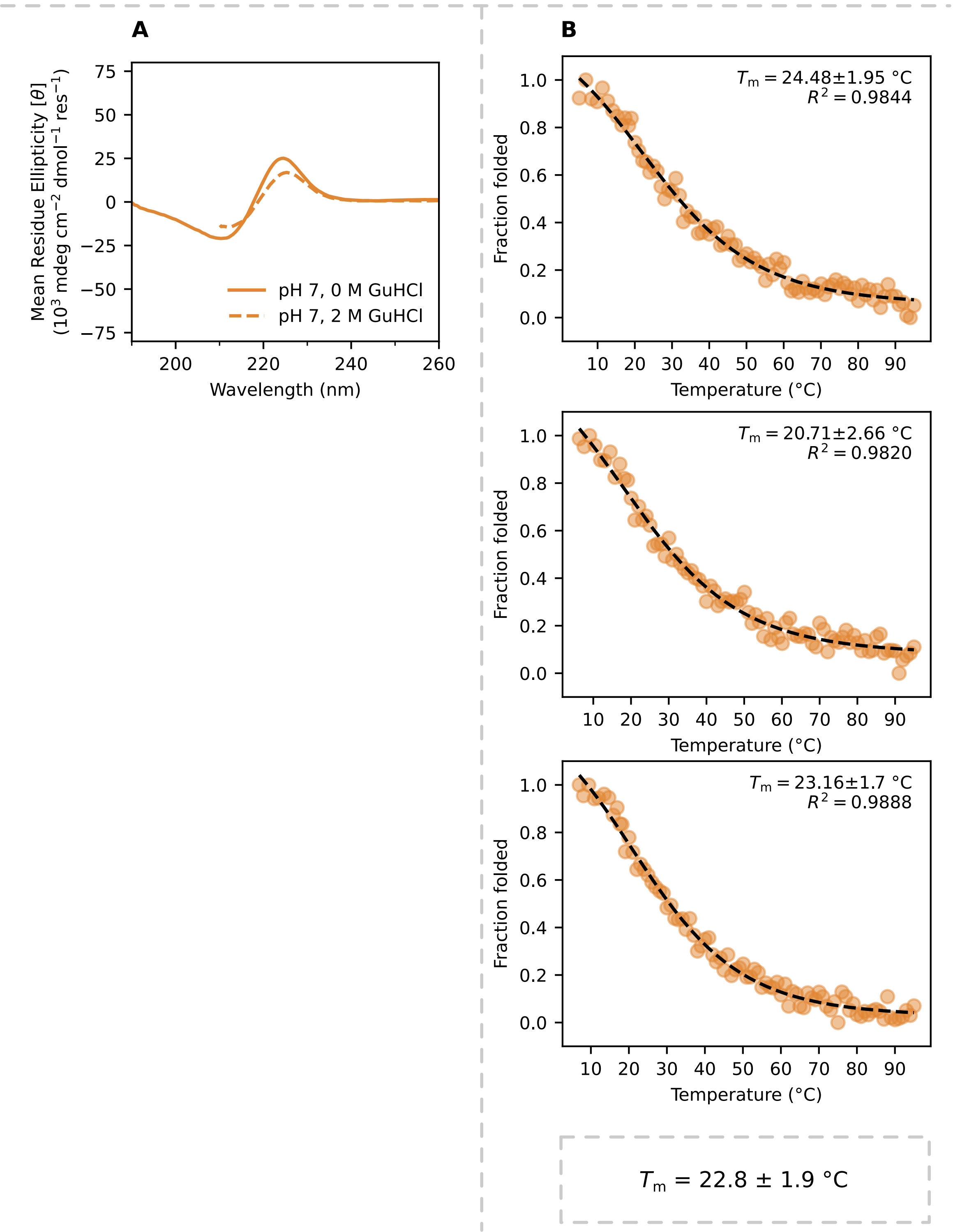

Figure S1 (continued). See complete caption on page 2.

Peptide **5**

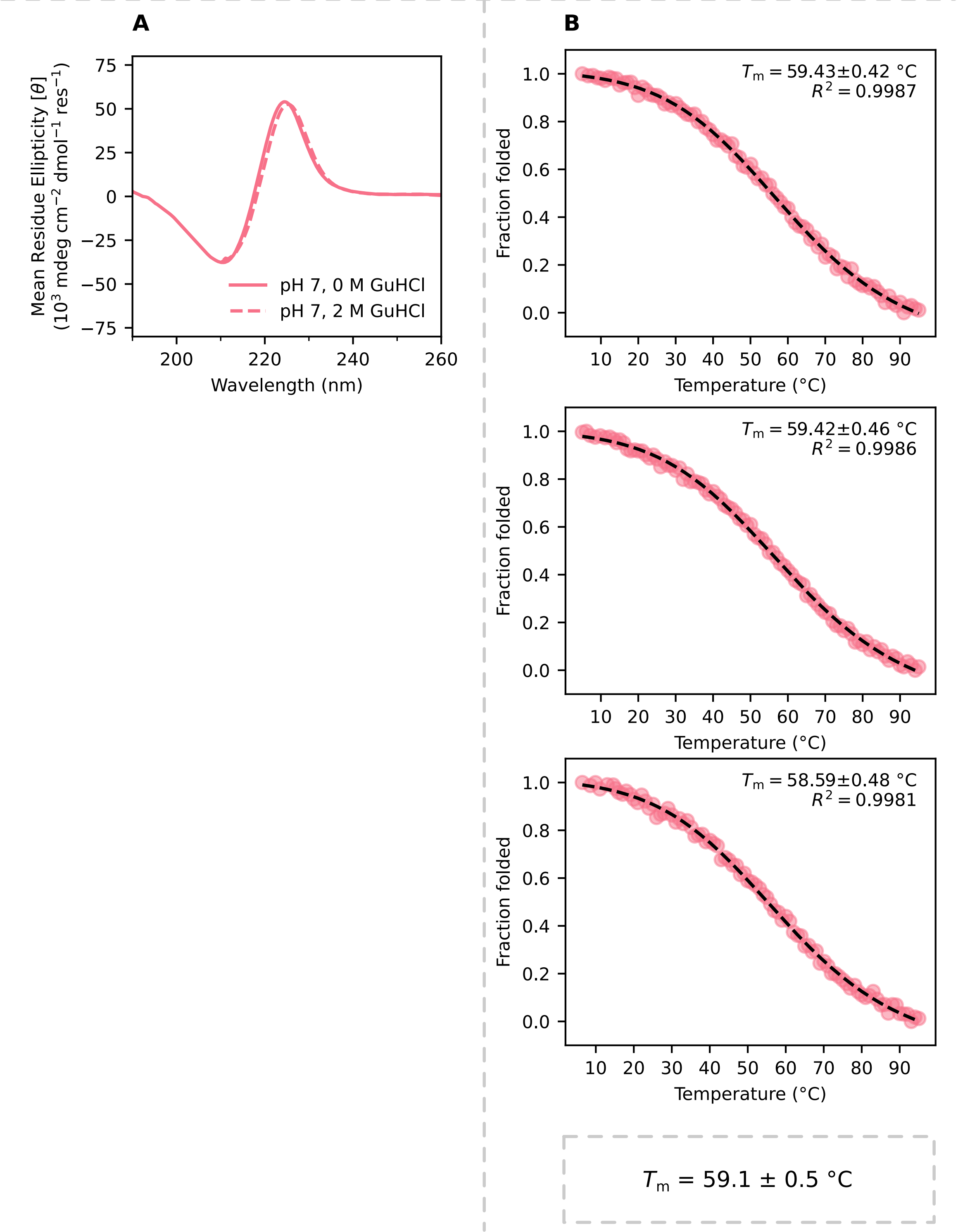

Figure S1 (continued). See complete caption on page 2.

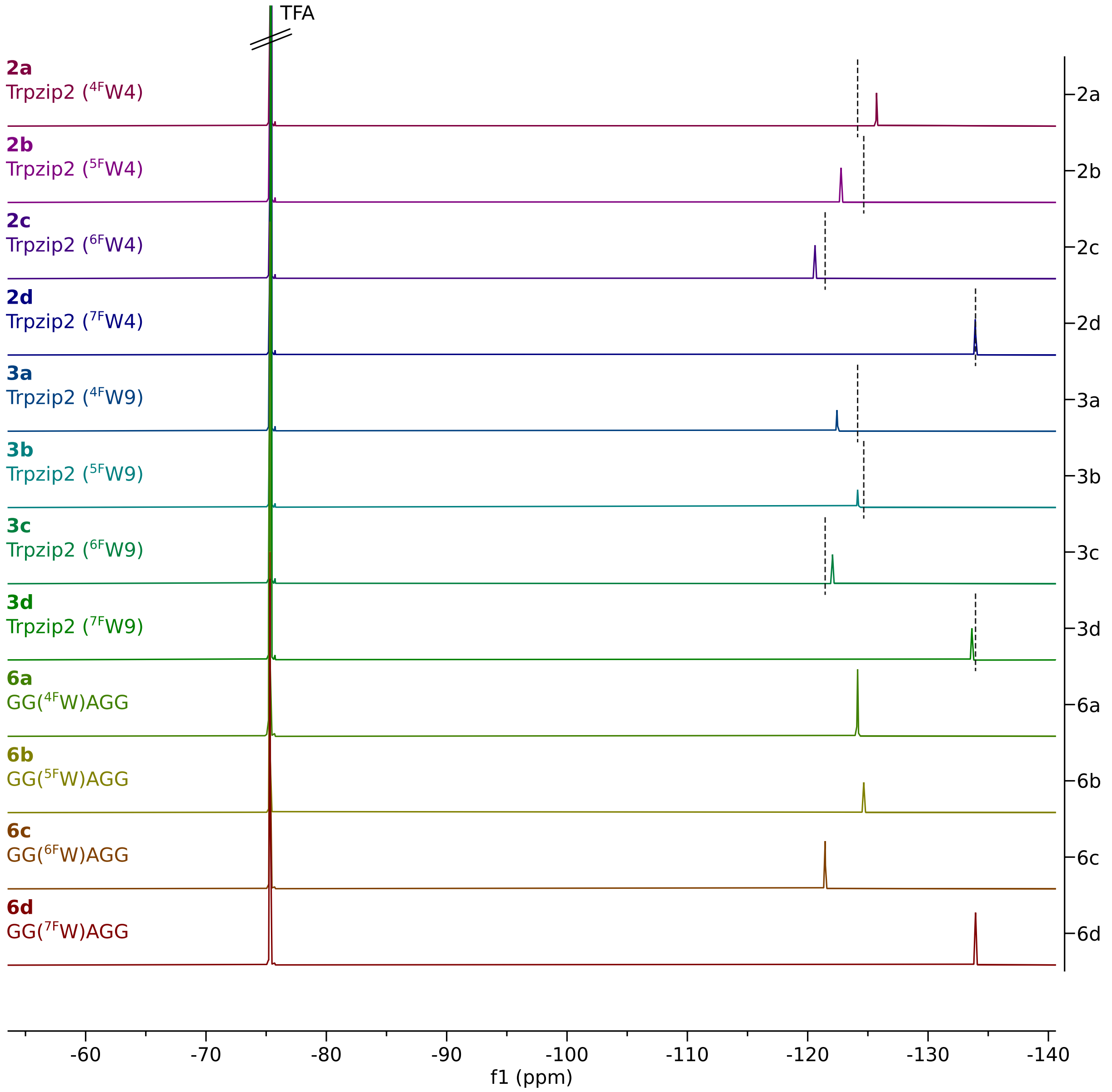

Figure S2. ^{19}F-NMR spectra of fluoro-Trp-substituted Trpzip2 analogues **2a**–**3d** recorded in water containing 10 % D_2O and fluoro-Trp random coil peptides **6a**–**6d** recorded in 50 mM phosphate buffer (pH 5.8) containing 1.0 M urea, and 10 % D_2O. The corresponding random coil chemical shifts of ^{4F}Trp, ^{5F}Trp, ^{6F}Trp, and ^{7F}Trp are indicated with dashed lines.

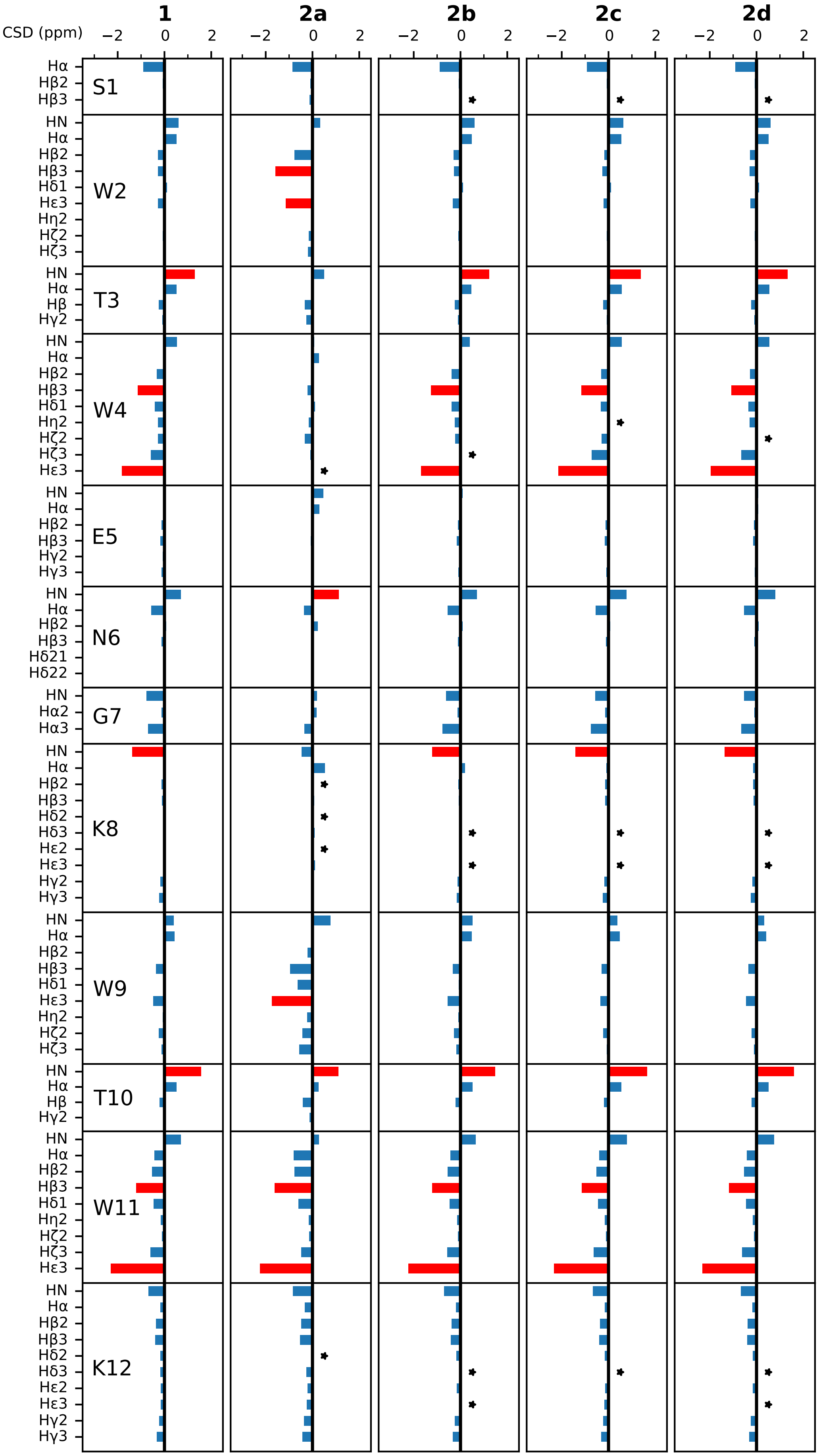

Figure S3. ^{1}H chemical shift deviations of protons in peptides **1**, and **2a–2d** against the respective random coil values. Random coil values of canonical amino acids were reported by Wishart *et al.*[2] Random coil values of fluoro-Trp residues are given in Table S10. Chemical shifts with deviations larger ±1.00 ppm are highlighted red, unassigned chemical shifts are indicated with an asterisk (*).

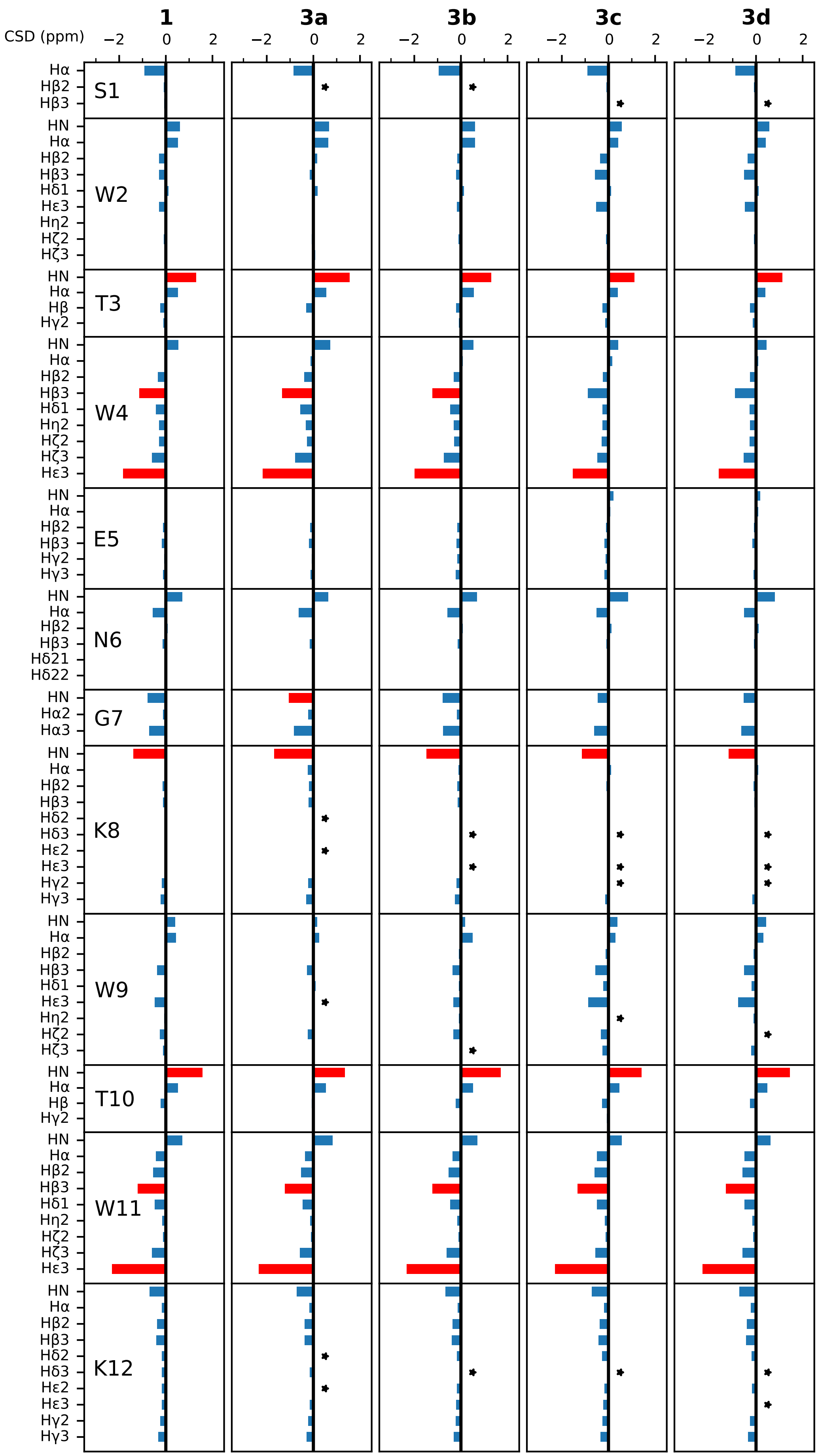

Figure S3 (continued). See complete caption on page 14.

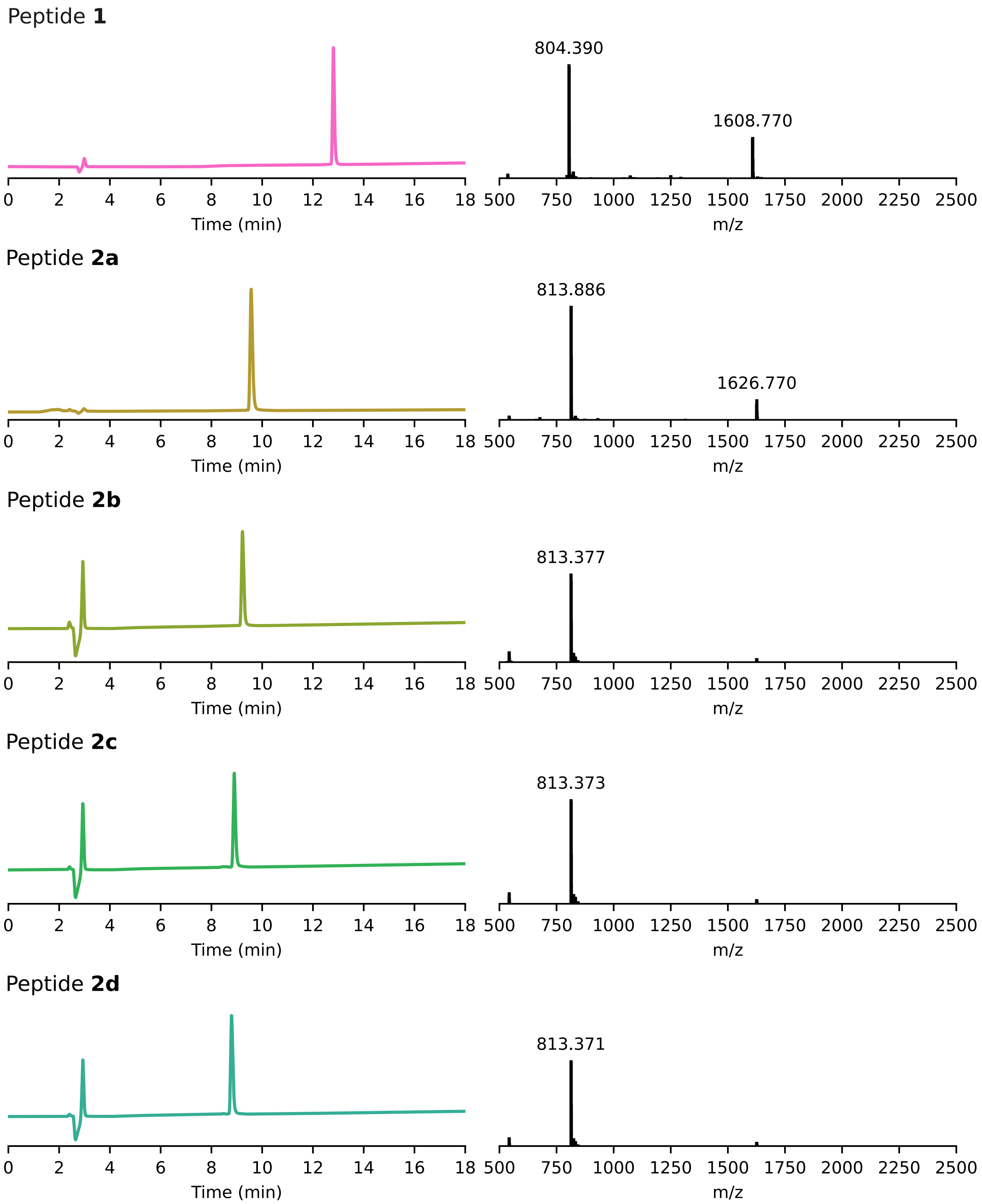

Figure S4. Analytical HPLC chromatograms (l.) and ESI-TOF mass spectra (r.) of purified peptides. Peptides **1**–**5** were analyzed using a linear gradient of 20 – 50 %B over 18 min, 1.00 mL min^{-1}. Peptides **6a**–**6d** were analyzed using a gradient of 5 %B over 3 min, 5 – 60 %B over 20 min, 1.00 mL min^{-1}.

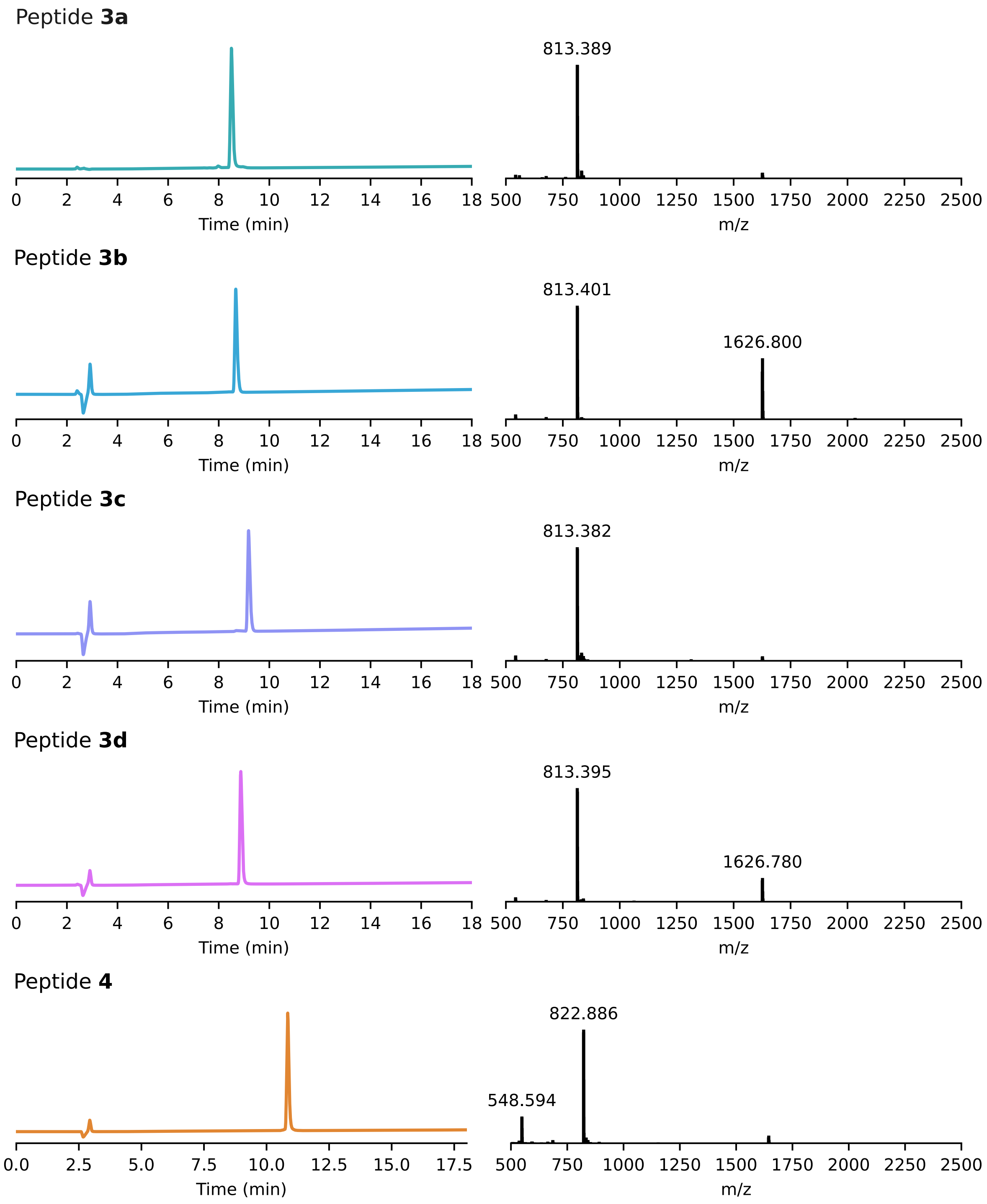

Figure S4 (continued). See complete caption on page 16.

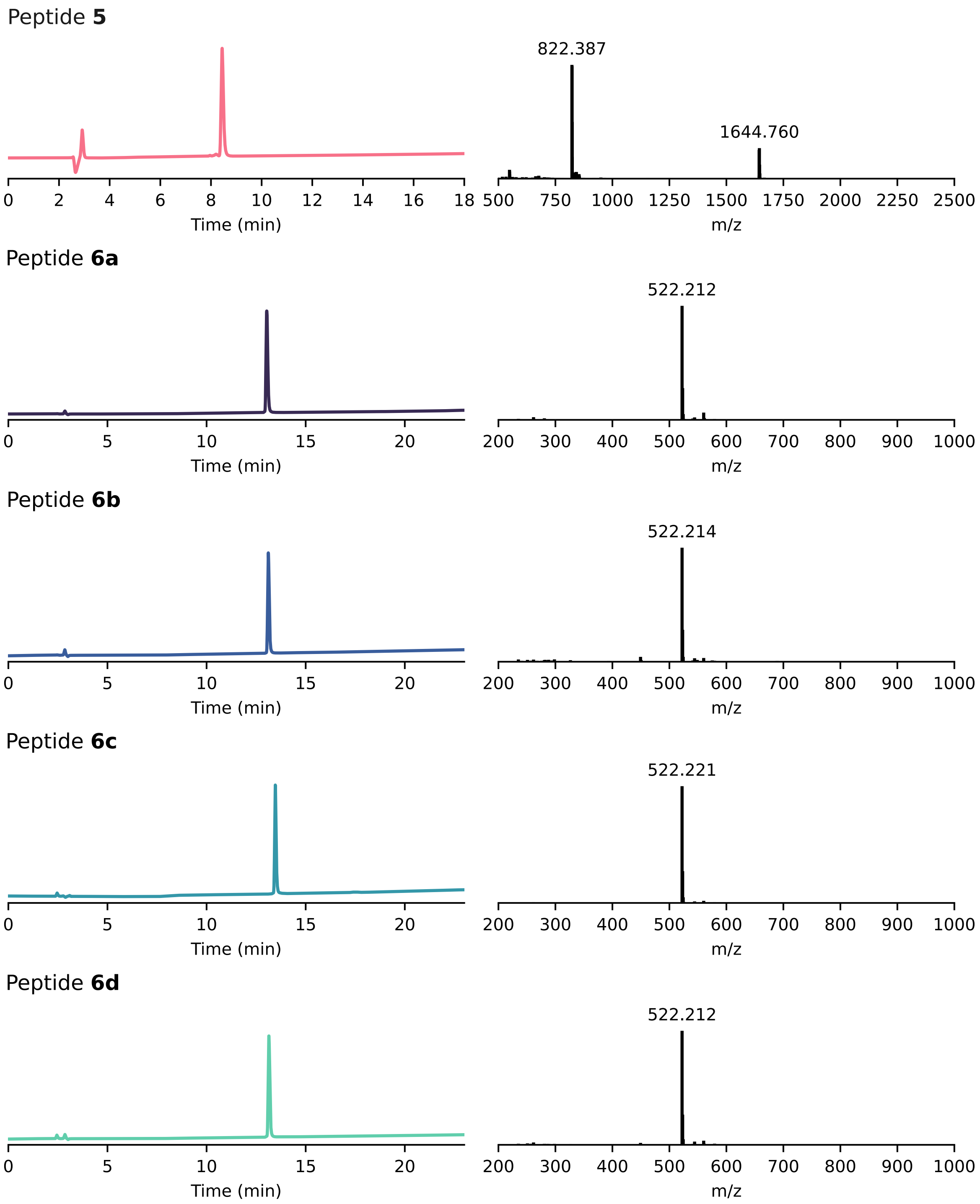

Figure S4 (continued). See complete caption on page 16.

2 Supplementary Tables

Table S1. Chemical shifts (ppm) of peptide **1** in water containing 10 % D_2O.

Residue	H^N	N	H^α	C^α	H^β	C^β	Others
S1			3.56	57.3	3.81, 3.81	62.5	
W2	8.86	123.1	5.17	57.2	3.01, 2.98	31.1	$C^{\delta1}$ 127.7, $C^{\epsilon3}$ 120.2, $C^{\eta2}$ 124.8, $C^{\zeta2}$ 115.1, $C^{\zeta3}$ 122.6, $H^{\delta1}$ 7.38, $H^{\epsilon1}$ 10.26, $H^{\epsilon3}$ 7.37, $H^{\eta2}$ 7.28, $H^{\zeta2}$ 7.42, $H^{\zeta3}$ 7.20, $N^{\epsilon1}$ 129.8
T3	9.45	118.5	4.87		4.00	71.5	$C^{\gamma2}$ 20.9, $H^{\gamma2}$ 1.11
W4	8.78	128.8	4.65		2.95, 2.13	28.9	$C^{\delta1}$ 127.2, $C^{\epsilon3}$ 120.1, $C^{\eta2}$ 124.7, $C^{\zeta2}$ 113.8, $C^{\zeta3}$ 121.6, $H^{\delta1}$ 6.85, $H^{\epsilon1}$ 9.88, $H^{\epsilon3}$ 5.83, $H^{\eta2}$ 6.97, $H^{\zeta2}$ 7.21, $H^{\zeta3}$ 6.59, $N^{\epsilon1}$ 128.3
E5	8.48	126.7	4.38	55.0	1.94, 1.79	31.3	C^{γ} 33.6, $H^{\gamma2}$ 2.24, $H^{\gamma3}$ 2.19
N6	9.11	122.7	4.18	54.1	2.91, 2.62	37.5	$H^{\delta21}$ 7.57, $H^{\delta22}$ 6.88, $N^{\delta2}$ 113.2
G7	7.56	102.0	3.84, 3.25	45.8			
K8	6.91	117.7	4.28	54.6	1.71, 1.64	35.5	C^{δ} 29.2, C^{ϵ} 42.3, C^{γ} 23.6, $H^{\delta2}$ 1.64, $H^{\delta3}$ 1.64, $H^{\epsilon2}$ 3.00, $H^{\epsilon3}$ 3.00, $H^{\gamma2}$ 1.26, $H^{\gamma3}$ 1.22
W9	8.65	124.4	5.09	57.4	3.23, 2.90	29.8	$C^{\delta1}$ 127.9, $C^{\epsilon3}$ 120.1, $C^{\eta2}$ 124.6, $C^{\zeta2}$ 115.2, $C^{\zeta3}$ 122.1, $H^{\delta1}$ 7.23, $H^{\epsilon1}$ 9.84, $H^{\epsilon3}$ 7.17, $H^{\eta2}$ 7.17, $H^{\zeta2}$ 7.25, $H^{\zeta3}$ 7.06, $N^{\epsilon1}$ 129.4
T10	9.72	122.0	4.87		4.03	71.9	$C^{\gamma2}$ 20.9, $H^{\gamma2}$ 1.18
W11	8.96	129.1	4.24	57.0	2.75, 2.06	28.6	$C^{\delta1}$ 127.8, $C^{\epsilon3}$ 120.8, $C^{\eta2}$ 124.0, $C^{\zeta2}$ 114.7, $C^{\zeta3}$ 120.5, $H^{\delta1}$ 6.80, $H^{\epsilon1}$ 10.00, $H^{\epsilon3}$ 5.35, $H^{\eta2}$ 7.09, $H^{\zeta2}$ 7.38, $H^{\zeta3}$ 6.58, $N^{\epsilon1}$ 129.9
K12	7.60	128.6	4.15	54.3	1.47, 1.35	33.6	C^{δ} 28.8, C^{ϵ} 42.0, C^{γ} 24.4, $H^{\delta2}$ 1.51, $H^{\delta3}$ 1.51, $H^{\epsilon2}$ 2.83, $H^{\epsilon3}$ 2.83, $H^{\gamma2}$ 1.21, $H^{\gamma3}$ 1.12
Amide13	7.35, 6.66	109.8					

Table S2. Chemical shifts (ppm) of peptide **2a** in water containing 10 % D_2O.

Residue	H^N	N	H^α	C^α	H^β	C^β	Others
S1			3.61	57.3	3.80, 3.75	62.5	
W2	8.58	123.0	4.70	56.0	2.51, 1.69	31.1	$C^{\delta1}$ 127.0, $C^{\varepsilon3}$ 121.0, $C^{\eta2}$ 124.5, $C^{\zeta2}$ 114.2, $C^{\zeta3}$ 122.4, $H^{\delta1}$ 7.32, $H^{\varepsilon1}$ 10.19, $H^{\varepsilon3}$ 6.50, $H^{\eta2}$ 7.20, $H^{\zeta2}$ 7.35, $H^{\zeta3}$ 6.99, $N^{\varepsilon1}$ 129.1
T3	8.64	116.5	4.42	60.3	3.91	71.1	$C^{\gamma2}$ 20.2, $H^{\gamma2}$ 0.94
^{4F}W4	8.34	124.1	4.97	58.0	3.38, 3.08	30.8	$C^{\delta1}$ 127.9, $C^{\eta2}$ 125.1, $C^{\zeta2}$ 111.1, $C^{\zeta3}$ 106.6, $H^{\delta1}$ 7.31, $H^{\varepsilon1}$ 10.26, $H^{\eta2}$ 7.00, $H^{\zeta2}$ 6.97, $H^{\zeta3}$ 6.74, $N^{\varepsilon1}$ 132.5, $F^{\varepsilon3}$ 125.7
E5	8.88	125.1	4.64	55.2	2.09, 1.88	30.7	C^{γ} 33.6, $H^{\gamma2}$ 2.31, $H^{\gamma3}$ 2.25
N6	9.52	125.4	4.38	54.3	3.07, 2.77	37.8	$H^{\delta21}$ 7.66, $H^{\delta22}$ 6.94, $N^{\delta2}$ 113.2
G7	8.53	103.1	4.13, 3.61	45.7			
K8	7.82	119.8	4.85		1.82	35.1	C^{δ} 29.1, C^{ε} 42.3, C^{γ} 24.6, $H^{\delta3}$ 1.77, $H^{\varepsilon3}$ 3.10, $H^{\gamma2}$ 1.48, $H^{\gamma3}$ 1.43
W9	9.02		4.68	57.3	3.08, 2.32	29.4	$C^{\delta1}$ 127.6, $C^{\varepsilon3}$ 120.3, $C^{\eta2}$ 124.3, $C^{\zeta2}$ 114.8, $C^{\zeta3}$ 121.0, $H^{\delta1}$ 6.63, $H^{\varepsilon1}$ 9.02, $H^{\varepsilon3}$ 5.91, $H^{\eta2}$ 7.02, $H^{\zeta2}$ 7.07, $H^{\zeta3}$ 6.61
T10	9.27	125.6	4.61	62.8	3.82	72.1	$C^{\gamma2}$ 20.4, $H^{\gamma2}$ 1.09
W11	8.53	128.7	3.85	56.7	2.52, 1.65	28.2	$C^{\delta1}$ 127.7, $C^{\varepsilon3}$ 120.8, $C^{\eta2}$ 124.0, $C^{\zeta2}$ 114.7, $C^{\zeta3}$ 120.5, $H^{\delta1}$ 6.66, $H^{\varepsilon1}$ 9.93, $H^{\varepsilon3}$ 5.41, $H^{\eta2}$ 7.09, $H^{\zeta2}$ 7.35, $H^{\zeta3}$ 6.70, $N^{\varepsilon1}$ 129.8
K12	7.46	128.5	4.00	54.2	1.36, 1.21	33.3	C^{δ} 28.7, C^{ε} 41.8, C^{γ} 24.2, $H^{\delta3}$ 1.42, $H^{\varepsilon2}$ 2.78, $H^{\varepsilon3}$ 2.74, $H^{\gamma2}$ 1.08, $H^{\gamma3}$ 1.01
Amide13	7.14, 6.54	109.6					

Table S3. Chemical shifts (ppm) of peptide **2b** in water containing 10 % D_2O.

Residue	H^N	N	H^α	C^α	H^β	C^β	Others
S1			3.58	57.3	3.82	62.5	
W2	8.86	123.2	5.14	57.3	2.99, 2.99	31.1	$C^{\delta1}$ 127.7, $C^{\varepsilon3}$ 120.2, $C^{\eta2}$ 124.8, $C^{\zeta2}$ 115.1, $C^{\zeta3}$ 122.6, $H^{\delta1}$ 7.38, $H^{\varepsilon1}$ 10.27, $H^{\varepsilon3}$ 7.33, $H^{\eta2}$ 7.28, $H^{\zeta2}$ 7.41, $H^{\zeta3}$ 7.20, $N^{\varepsilon1}$ 129.8
T3	9.38	118.5	4.82		3.99	71.5	$C^{\gamma2}$ 20.9, $H^{\gamma2}$ 1.10
^{5F}W4	8.67	128.6	4.65	56.3	2.85, 1.95	28.6	$C^{\delta1}$ 129.5, $C^{\varepsilon3}$ 104.9, $C^{\eta2}$ 112.9, $C^{\zeta2}$ 114.8, $H^{\delta1}$ 6.93, $H^{\varepsilon1}$ 10.01, $H^{\varepsilon3}$ 5.61, $H^{\eta2}$ 6.77, $H^{\zeta2}$ 7.21, $N^{\varepsilon1}$ 128.8, $F^{\zeta3}$ 122.8
E5	8.52	126.1	4.34	55.0	1.94, 1.79	31.0	C^{γ} 33.1, $H^{\gamma2}$ 2.25, $H^{\gamma3}$ 2.21
N6	9.11	122.5	4.19	54.1	2.91, 2.63	37.6	$H^{\delta21}$ 7.57, $H^{\delta22}$ 6.88, $N^{\delta2}$ 113.1
G7	7.71	102.3	3.84, 3.19	45.8			
K8	7.07	117.8	4.51	54.6	1.75, 1.68	35.7	C^{δ} 29.2, C^{ε} 42.3, C^{γ} 23.8, $H^{\delta2}$ 1.67, $H^{\varepsilon2}$ 3.01, $H^{\gamma2}$ 1.31, $H^{\gamma3}$ 1.27
W9	8.77	124.4	5.14	57.3	3.23, 2.93	29.8	$C^{\delta1}$ 128.6, $C^{\varepsilon3}$ 120.0, $C^{\eta2}$ 124.5, $C^{\zeta2}$ 115.0, $C^{\zeta3}$ 122.0, $H^{\delta1}$ 7.20, $H^{\varepsilon1}$ 9.81, $H^{\varepsilon3}$ 7.10, $H^{\eta2}$ 7.16, $H^{\zeta2}$ 7.22, $H^{\zeta3}$ 7.00, $N^{\varepsilon1}$ 129.4
T10	9.63	121.7	4.87	61.9	4.02	71.9	$C^{\gamma2}$ 20.9, $H^{\gamma2}$ 1.18
W11	8.91	128.8	4.23	57.0	2.75, 2.06	28.7	$C^{\delta1}$ 127.8, $C^{\varepsilon3}$ 120.8, $C^{\eta2}$ 124.0, $C^{\zeta2}$ 114.7, $C^{\zeta3}$ 120.6, $H^{\delta1}$ 6.80, $H^{\varepsilon1}$ 10.00, $H^{\varepsilon3}$ 5.42, $H^{\eta2}$ 7.10, $H^{\zeta2}$ 7.39, $H^{\zeta3}$ 6.62, $N^{\varepsilon1}$ 130.0
K12	7.59	128.6	4.13	54.4	1.46, 1.34	33.7	C^{δ} 28.9, C^{ε} 42.0, C^{γ} 24.5, $H^{\delta2}$ 1.50, $H^{\varepsilon2}$ 2.83, $H^{\gamma2}$ 1.19, $H^{\gamma3}$ 1.11
Amide13	7.34, 6.67	109.9					

Table S4. Chemical shifts (ppm) of peptide **2c** in water containing 10 % D_2O.

Residue	H^N	N	H^α	C^α	H^β	C^β	Others
S1			3.55	57.3	3.81	62.5	
W2	8.88	123.0	5.21	57.2	3.12, 3.01	31.1	$C^{\delta1}$ 127.8, $C^{\varepsilon3}$ 120.1, $C^{\eta2}$ 124.8, $C^{\zeta2}$ 115.2, $C^{\zeta3}$ 122.6, $H^{\delta1}$ 7.38, $H^{\varepsilon1}$ 10.28, $H^{\varepsilon3}$ 7.45, $H^{\eta2}$ 7.29, $H^{\zeta2}$ 7.42, $H^{\zeta3}$ 7.22, $N^{\varepsilon1}$ 129.9
T3	9.54	118.6	4.92	60.9	4.01	71.7	$C^{\gamma2}$ 21.0, $H^{\gamma2}$ 1.13
^{6F}W4	8.85	129.1	4.64	56.3	2.93, 2.08	28.7	$C^{\delta1}$ 127.6, $C^{\varepsilon3}$ 120.9, $C^{\zeta2}$ 100.0, $C^{\zeta3}$ 109.8, $H^{\delta1}$ 6.92, $H^{\varepsilon1}$ 9.92, $H^{\varepsilon3}$ 5.42, $H^{\zeta2}$ 6.91, $H^{\zeta3}$ 6.23, $N^{\varepsilon1}$ 129.2, $F^{\eta2}$ 120.6
E5	8.47	126.9	4.39	54.9	1.94, 1.79	31.3	C^{γ} 33.3, $H^{\gamma2}$ 2.26, $H^{\gamma3}$ 2.21
N6	9.16	122.8	4.19	54.0	2.90, 2.63	37.5	$H^{\delta21}$ 7.57, $H^{\delta22}$ 6.88, $N^{\delta2}$ 113.2
G7	7.76	101.9	3.81, 3.20	45.7			
K8	6.87	117.0	4.23	54.5	1.69, 1.61	35.7	C^{δ} 29.2, C^{ε} 42.3, C^{γ} 23.5, $H^{\delta2}$ 1.64, $H^{\varepsilon2}$ 3.00, $H^{\gamma2}$ 1.26, $H^{\gamma3}$ 1.19
W9	8.63	123.7	5.15	57.4	3.26, 2.96	29.9	$C^{\delta1}$ 127.9, $C^{\varepsilon3}$ 120.0, $C^{\eta2}$ 124.7, $C^{\zeta2}$ 115.3, $C^{\zeta3}$ 122.2, $H^{\delta1}$ 7.29, $H^{\varepsilon1}$ 9.95, $H^{\varepsilon3}$ 7.31, $H^{\eta2}$ 7.19, $H^{\zeta2}$ 7.27, $H^{\zeta3}$ 7.10, $N^{\varepsilon1}$ 129.6
T10	9.80	122.0	4.89		4.04	71.9	$C^{\gamma2}$ 20.8, $H^{\gamma2}$ 1.19
W11	9.03	129.3	4.27	57.1	2.77, 2.12	28.7	$C^{\delta1}$ 127.8, $C^{\varepsilon3}$ 120.8, $C^{\eta2}$ 124.0, $C^{\zeta2}$ 114.7, $C^{\zeta3}$ 120.5, $H^{\delta1}$ 6.82, $H^{\varepsilon1}$ 10.00, $H^{\varepsilon3}$ 5.32, $H^{\eta2}$ 7.09, $H^{\zeta2}$ 7.39, $H^{\zeta3}$ 6.55, $N^{\varepsilon1}$ 130.0
K12	7.62	128.7	4.15	54.3	1.47, 1.35	33.7	C^{δ} 28.9, C^{ε} 42.0, C^{γ} 24.5, $H^{\delta2}$ 1.51, $H^{\varepsilon2}$ 2.85, $H^{\varepsilon3}$ 2.81, $H^{\gamma2}$ 1.21, $H^{\gamma3}$ 1.13
Amide13	6.68, 7.38	109.9					

Table S5. Chemical shifts (ppm) of peptide **2d** in water containing 10 % D_2O.

Residue	H^N	N	H^α	C^α	H^β	C^β	Others
S1			3.56	54.6	3.81	59.7	
W2	8.86	122.9	5.18	54.4	3.01, 2.97	28.4	$C^{\delta1}$ 125.0, $C^{\varepsilon3}$ 117.4, $C^{\eta2}$ 122.1, $C^{\zeta2}$ 112.4, $C^{\zeta3}$ 119.9, $H^{\delta1}$ 7.37, $H^{\varepsilon1}$ 10.28, $H^{\varepsilon3}$ 7.38, $H^{\eta2}$ 7.28, $H^{\zeta2}$ 7.42, $H^{\zeta3}$ 7.20, $N^{\varepsilon1}$ 129.8
T3	9.47	118.5	4.89	58.1	4.01	69.0	$C^{\gamma2}$ 18.2, $H^{\gamma2}$ 1.12
^{7F}W4	8.85	128.6	4.66		2.99, 2.19	26.0	$C^{\delta1}$ 125.3, $C^{\varepsilon3}$ 117.5, $C^{\eta2}$ 106.8, $C^{\zeta3}$ 119.2, $H^{\delta1}$ 6.95, $H^{\varepsilon1}$ 10.36, $H^{\varepsilon3}$ 5.46, $H^{\eta2}$ 6.69, $H^{\zeta3}$ 6.43, $N^{\varepsilon1}$ 122.7, $F^{\zeta2}$ 133.9
E5	8.50	126.7	4.43	52.2	1.96, 1.81	28.5	C^{γ} 30.5, $H^{\gamma2}$ 2.27, $H^{\gamma3}$ 2.23
N6	9.20	122.8	4.21	51.3	2.92, 2.65	34.7	$H^{\delta21}$ 7.58, $H^{\delta22}$ 6.89, $N^{\delta2}$ 113.2
G7	7.79	101.8	3.86, 3.30	42.8			
K8	6.92	117.3	4.18	51.9	1.69, 1.62	32.7	C^{δ} 26.5, C^{ε} 39.5, C^{γ} 20.7, $H^{\delta2}$ 1.64, $H^{\varepsilon2}$ 3.00, $H^{\gamma2}$ 1.26, $H^{\gamma3}$ 1.19
W9	8.58	123.9	5.07	54.6	3.25, 2.92	27.1	$C^{\delta1}$ 125.2, $C^{\varepsilon3}$ 117.3, $C^{\eta2}$ 121.9, $C^{\zeta2}$ 112.5, $C^{\zeta3}$ 119.3, $H^{\delta1}$ 7.25, $H^{\varepsilon1}$ 9.91, $H^{\varepsilon3}$ 7.20, $H^{\eta2}$ 7.19, $H^{\zeta2}$ 7.29, $H^{\zeta3}$ 7.06, $N^{\varepsilon1}$ 129.4
T10	9.76	122.1	4.87	59.2	4.03	69.2	$C^{\gamma2}$ 18.0, $H^{\gamma2}$ 1.18
W11	9.00	129.3	4.24	54.3	2.76, 2.10	26.0	$C^{\delta1}$ 125.1, $C^{\varepsilon3}$ 118.1, $C^{\eta2}$ 121.2, $C^{\zeta2}$ 112.0, $C^{\zeta3}$ 117.8, $H^{\delta1}$ 6.81, $H^{\varepsilon1}$ 10.00, $H^{\varepsilon3}$ 5.33, $H^{\eta2}$ 7.09, $H^{\zeta2}$ 7.39, $H^{\zeta3}$ 6.56, $N^{\varepsilon1}$ 129.9
K12	7.61	128.7	4.15	51.6	1.47, 1.34	30.9	C^{δ} 26.1, C^{ε} 39.3, C^{γ} 21.7, $H^{\delta2}$ 1.51, $H^{\varepsilon2}$ 2.83, $H^{\gamma2}$ 1.20, $H^{\gamma3}$ 1.12
Amide13	7.36, 6.67	110.0					

Table S6. Chemical shifts (ppm) of peptide **3a** in water containing 10 % D_2O.

Residue	H^N	N	H^α	C^α	H^β	C^β	Others
S1			3.62	57.3	3.83	62.5	
W2	8.91	123.6	5.29	57.3	3.46, 3.12	30.0	$C^{\delta1}$ 127.8, $C^{\varepsilon3}$ 120.3, $C^{\eta2}$ 124.9, $C^{\zeta2}$ 115.2, $C^{\zeta3}$ 122.7, $H^{\delta1}$ 7.45, $H^{\varepsilon1}$ 10.30, $H^{\varepsilon3}$ 7.61, $H^{\eta2}$ 7.31, $H^{\zeta2}$ 7.43, $H^{\zeta3}$ 7.25, $N^{\varepsilon1}$ 129.9
T3	9.70	120.7	4.90	61.3	3.93	71.9	$C^{\gamma2}$ 21.1, $H^{\gamma2}$ 1.17
W4	8.96	130.1	4.54	56.9	2.90, 1.93	28.3	$C^{\delta1}$ 127.1, $C^{\varepsilon3}$ 119.9, $C^{\eta2}$ 124.8, $C^{\zeta2}$ 113.6, $C^{\zeta3}$ 121.3, $H^{\delta1}$ 6.71, $H^{\varepsilon1}$ 9.84, $H^{\varepsilon3}$ 5.48, $H^{\eta2}$ 6.93, $H^{\zeta2}$ 7.23, $H^{\zeta3}$ 6.41, $N^{\varepsilon1}$ 128.0
E5	8.37	127.9	4.33	55.0	1.93, 1.78	31.4	C^{γ} 33.4, $H^{\gamma2}$ 2.23, $H^{\gamma3}$ 2.19
N6	9.03	121.9	4.12	54.0	2.87, 2.59	37.5	$H^{\delta21}$ 7.56, $H^{\delta22}$ 6.86, $N^{\delta2}$ 113.3
G7	7.27	101.5	3.74, 3.13	45.9			
K8	6.61	116.9	4.09	54.5	1.65, 1.55	35.6	C^{δ} 29.2, C^{ε} 42.3, C^{γ} 23.4, $H^{\delta3}$ 1.62, $H^{\varepsilon3}$ 2.98, $H^{\gamma2}$ 1.22, $H^{\gamma3}$ 1.14
^{4F}W9	8.43	123.0	4.94	59.1	3.39, 3.01	30.6	$C^{\delta1}$ 128.1, $C^{\eta2}$ 125.2, $C^{\zeta2}$ 111.5, $C^{\zeta3}$ 106.9, $H^{\delta1}$ 7.28, $H^{\varepsilon1}$ 10.22, $H^{\eta2}$ 7.10, $H^{\zeta2}$ 7.05, $H^{\zeta3}$ 6.88, $N^{\varepsilon1}$ 132.2, $F^{\varepsilon3}$ 122.4
T10	9.49	122.1	4.89	62.1	4.17	71.8	$C^{\gamma2}$ 21.0, $H^{\gamma2}$ 1.20
W11	9.07	129.2	4.31	56.9	2.76, 2.05	28.6	$C^{\delta1}$ 127.8, $C^{\varepsilon3}$ 120.9, $C^{\eta2}$ 124.0, $C^{\zeta2}$ 114.8, $C^{\zeta3}$ 120.4, $H^{\delta1}$ 6.81, $H^{\varepsilon1}$ 10.01, $H^{\varepsilon3}$ 5.30, $H^{\eta2}$ 7.11, $H^{\zeta2}$ 7.40, $H^{\zeta3}$ 6.59, $N^{\varepsilon1}$ 129.9
K12	7.58	128.6	4.15	54.2	1.47, 1.37	33.7	C^{δ} 28.8, C^{ε} 42.0, C^{γ} 24.4, $H^{\delta3}$ 1.52, $H^{\varepsilon3}$ 2.83, $H^{\gamma2}$ 1.22, $H^{\gamma3}$ 1.15
Amide13	7.36, 6.65	109.6					

Table S7. Chemical shifts (ppm) of peptide **3b** in water containing 10 % D_2O.

Residue	H^N	N	H^α	C^α	H^β	C^β	Others
S1			3.52	57.3	3.81	62.5	
W2	8.85	123.0	5.26	57.4	3.14, 3.07	31.3	$C^{\delta1}$ 127.8, $C^{\varepsilon3}$ 120.2, $C^{\eta2}$ 124.8, $C^{\zeta2}$ 115.1, $C^{\zeta3}$ 122.6, $H^{\delta1}$ 7.40, $H^{\varepsilon1}$ 10.25, $H^{\varepsilon3}$ 7.48, $H^{\eta2}$ 7.28, $H^{\zeta2}$ 7.40, $H^{\zeta3}$ 7.18, $N^{\varepsilon1}$ 129.9
T3	9.45	117.5	4.90	60.9	4.03	71.8	$C^{\gamma2}$ 21.2, $H^{\gamma2}$ 1.12
W4	8.78	128.0	4.74		2.98, 2.05	29.0	$C^{\delta1}$ 127.9, $C^{\varepsilon3}$ 120.0, $C^{\eta2}$ 124.7, $C^{\zeta2}$ 113.7, $C^{\zeta3}$ 121.4, $H^{\delta1}$ 6.81, $H^{\varepsilon1}$ 9.86, $H^{\varepsilon3}$ 5.66, $H^{\eta2}$ 6.94, $H^{\zeta2}$ 7.21, $H^{\zeta3}$ 6.45, $N^{\varepsilon1}$ 128.2
E5	8.40	127.0	4.35	55.2	1.90, 1.76	32.1	C^{γ} 35.0, $H^{\gamma2}$ 2.16, $H^{\gamma3}$ 2.09
N6	9.09	122.8	4.16	54.0	2.90, 2.61	37.5	$H^{\delta21}$ 7.57, $H^{\delta22}$ 6.88, $N^{\delta2}$ 113.4
G7	7.54	101.8	3.79, 3.19	45.8			
K8	6.81	117.5	4.21	54.6	1.69, 1.61	35.5	C^{δ} 29.2, C^{ε} 42.3, C^{γ} 23.5, $H^{\delta2}$ 1.63, $H^{\varepsilon2}$ 2.99, $H^{\gamma2}$ 1.26, $H^{\gamma3}$ 1.18
^{5F}W9	8.45	123.5	5.15	57.1	3.15, 2.86	29.8	$C^{\delta1}$ 129.6, $C^{\varepsilon3}$ 104.8, $C^{\eta2}$ 112.7, $C^{\zeta2}$ 116.0, $H^{\delta1}$ 7.23, $H^{\varepsilon1}$ 9.85, $H^{\varepsilon3}$ 6.98, $H^{\eta2}$ 6.93, $H^{\zeta2}$ 7.12, $N^{\varepsilon1}$ 129.2, $F^{\zeta3}$ 124.2
T10	9.86	121.8	4.87	61.9	4.01	71.9	$C^{\gamma2}$ 20.9, $H^{\gamma2}$ 1.17
W11	8.95	129.1	4.30	56.8	2.76, 2.05	28.8	$C^{\delta1}$ 127.3, $C^{\varepsilon3}$ 120.8, $C^{\eta2}$ 124.0, $C^{\zeta2}$ 114.8, $C^{\zeta3}$ 120.6, $H^{\delta1}$ 6.81, $H^{\varepsilon1}$ 10.01, $H^{\varepsilon3}$ 5.33, $H^{\eta2}$ 7.10, $H^{\zeta2}$ 7.39, $H^{\zeta3}$ 6.57, $N^{\varepsilon1}$ 130.0
K12	7.63	128.4	4.18	54.1	1.48, 1.37	33.6	C^{δ} 28.7, C^{ε} 42.0, C^{γ} 24.5, $H^{\delta2}$ 1.50, $H^{\varepsilon2}$ 2.82, $H^{\varepsilon3}$ 2.78, $H^{\gamma2}$ 1.22, $H^{\gamma3}$ 1.14
Amide13	7.36, 6.66	109.5					

Table S8. Chemical shifts (ppm) of peptide **3c** in water containing 10 % D_2O.

Residue	H^N	N	H^α	C^α	H^β	C^β	Others
S1			3.57	57.4	3.80	62.6	
W2	8.81	123.0	5.08	57.2	2.92, 2.70	31.0	$C^{\delta1}$ 127.6, $C^{\varepsilon3}$ 120.3, $C^{\eta2}$ 124.7, $C^{\zeta2}$ 114.8, $C^{\zeta3}$ 122.6, $H^{\delta1}$ 7.38, $H^{\varepsilon1}$ 10.24, $H^{\varepsilon3}$ 7.12, $H^{\eta2}$ 7.25, $H^{\zeta2}$ 7.39, $H^{\zeta3}$ 7.12, $N^{\varepsilon1}$ 129.7
T3	9.26	118.0	4.75		3.99	71.6	$C^{\gamma2}$ 20.7, $H^{\gamma2}$ 1.06
W4	8.67	127.3	4.82		3.05, 2.38	29.1	$C^{\varepsilon3}$ 119.9, $C^{\eta2}$ 124.7, $C^{\zeta2}$ 114.1, $C^{\zeta3}$ 121.8, $H^{\delta1}$ 7.01, $H^{\varepsilon1}$ 9.95, $H^{\varepsilon3}$ 6.13, $H^{\eta2}$ 7.00, $H^{\zeta2}$ 7.21, $H^{\zeta3}$ 6.70
E5	8.62	126.4	4.43	55.3	1.95, 1.79	31.6	C^{γ} 34.6, $H^{\gamma2}$ 2.20, $H^{\gamma3}$ 2.14
N6	9.23	123.7	4.23	54.2	2.95, 2.66	37.6	$H^{\delta21}$ 7.59, $H^{\delta22}$ 6.89, $N^{\delta2}$ 113.3
G7	7.87	102.3	3.92, 3.35	45.8			
K8	7.16	118.1	4.44	54.8	1.75, 1.69	35.4	C^{δ} 29.1, C^{ε} 42.3, C^{γ} 23.7, $H^{\delta2}$ 1.68, $H^{\varepsilon2}$ 3.03, $H^{\gamma3}$ 1.29
^{6F}W9	8.67	125.7	4.95	57.2	3.13, 2.69	29.6	$C^{\varepsilon3}$ 120.9, $C^{\zeta2}$ 101.0, $C^{\zeta3}$ 110.0, $H^{\delta1}$ 7.02, $H^{\varepsilon1}$ 9.75, $H^{\varepsilon3}$ 6.71, $H^{\zeta2}$ 6.88, $H^{\zeta3}$ 6.71, $F^{\eta2}$ 122.1
T10	9.57	122.6	4.81		3.96	72.1	$C^{\gamma2}$ 20.7, $H^{\gamma2}$ 1.14
W11	8.82	128.8	4.16	56.9	2.70, 1.94	28.5	$C^{\delta1}$ 127.8, $C^{\varepsilon3}$ 120.8, $C^{\eta2}$ 124.0, $C^{\zeta2}$ 114.8, $C^{\zeta3}$ 120.5, $H^{\delta1}$ 6.77, $H^{\varepsilon1}$ 9.98, $H^{\varepsilon3}$ 5.36, $H^{\eta2}$ 7.09, $H^{\zeta2}$ 7.38, $H^{\zeta3}$ 6.62, $N^{\varepsilon1}$ 129.9
K12	7.57	128.4	4.13	54.2	1.46, 1.32	33.5	C^{δ} 28.6, C^{ε} 42.0, C^{γ} 24.3, $H^{\delta2}$ 1.41, $H^{\varepsilon2}$ 2.82, $H^{\varepsilon3}$ 2.77, $H^{\gamma2}$ 1.18, $H^{\gamma3}$ 1.10
Amide13	7.29, 6.62	109.6					

Table S9. Chemical shifts (ppm) of peptide **3d** in water containing 10 % D_2O.

Residue	H^N	N	H^α	C^α	H^β	C^β	Others
S1			3.58	57.4	3.81	62.6	
W2	8.82	123.0	5.08	57.2	2.94, 2.77	30.8	$C^{\delta1}$ 125.0, $C^{\varepsilon3}$ 117.6, $C^{\eta2}$ 122.1, $C^{\zeta2}$ 112.2, $C^{\zeta3}$ 119.7, $H^{\delta1}$ 7.38, $H^{\varepsilon1}$ 10.26, $H^{\varepsilon3}$ 7.17, $H^{\eta2}$ 7.26, $H^{\zeta2}$ 7.41, $H^{\zeta3}$ 7.16, $N^{\varepsilon1}$ 129.7
T3	9.29	118.1	4.74		3.99	71.6	$C^{\gamma2}$ 20.6, $H^{\gamma2}$ 1.07
W4	8.69	127.7	4.75		3.04, 2.37	29.1	$C^{\delta1}$ 124.7, $C^{\varepsilon3}$ 117.1, $C^{\eta2}$ 122.0, $C^{\zeta2}$ 111.3, $C^{\zeta3}$ 119.1, $H^{\delta1}$ 6.99, $H^{\varepsilon1}$ 9.94, $H^{\varepsilon3}$ 6.05, $H^{\eta2}$ 7.00, $H^{\zeta2}$ 7.22, $H^{\zeta3}$ 6.66, $N^{\varepsilon1}$ 128.6
E5	8.61	126.2	4.44	55.1	1.96, 1.81	31.1	C^{γ} 33.4, $H^{\gamma2}$ 2.27, $H^{\gamma3}$ 2.21
N6	9.21	123.3	4.22	54.2	2.94, 2.66	37.5	$H^{\delta21}$ 7.59, $H^{\delta22}$ 6.89, $N^{\delta2}$ 113.2
G7	7.81	102.2	3.91, 3.33	45.8			
K8	7.11	118.0	4.41	54.7	1.74, 1.68	35.4	C^{δ} 29.1, C^{ε} 42.3, C^{γ} 23.7, $H^{\delta2}$ 1.67, $H^{\varepsilon2}$ 3.02, $H^{\gamma3}$ 1.29
^{7F}W9	8.72	125.5	4.98	57.2	3.17, 2.75	29.6	$C^{\delta1}$ 126.0, $C^{\varepsilon3}$ 113.4, $C^{\eta2}$ 106.5, $H^{\delta1}$ 7.11, $H^{\varepsilon1}$ 10.15, $H^{\varepsilon3}$ 6.65, $H^{\eta2}$ 6.88, $H^{\zeta3}$ 6.88, $N^{\varepsilon1}$ 123.7, $F^{\zeta2}$ 133.6
T10	9.61	122.6	4.83		3.98	72.1	$C^{\gamma2}$ 20.6, $H^{\gamma2}$ 1.16
W11	8.88	129.0	4.16	57.0	2.71, 1.98	28.5	$C^{\delta1}$ 125.2, $C^{\varepsilon3}$ 118.1, $C^{\eta2}$ 121.3, $C^{\zeta2}$ 112.0, $C^{\zeta3}$ 117.8, $H^{\delta1}$ 6.78, $H^{\varepsilon1}$ 9.99, $H^{\varepsilon3}$ 5.36, $H^{\eta2}$ 7.09, $H^{\zeta2}$ 7.38, $H^{\zeta3}$ 6.61, $N^{\varepsilon1}$ 129.9
K12	7.58	128.6	4.10	54.4	1.44, 1.32	33.5	C^{δ} 28.8, C^{ε} 42.0, C^{γ} 24.3, $H^{\delta2}$ 1.49, $H^{\varepsilon2}$ 2.81, $H^{\gamma2}$ 1.18, $H^{\gamma3}$ 1.10
Amide13	7.32, 6.64	109.8					

Table S10. Random coil chemical shifts (ppm) of fluorinated Trp analogues determined from peptides **6a**–**6d** in 50 mM phosphate buffer (pH 5.8) containing 1.0 M urea, and 10 % D_2O according to Wishart *et al.*[2]

Residue	H^N	N	H^α	C^α	H^β	C^β	Others
^{4F}W	8.26	121.2	4.69	58.1	3.34, 3.28	30.9	$C^{\delta1}$ 127.9, $C^{\eta2}$ 125.3, $C^{\zeta2}$ 111.0, $C^{\zeta3}$ 107.0, $H^{\delta1}$ 7.20, $H^{\varepsilon1}$ 10.43, $H^{\eta2}$ 7.16, $H^{\zeta2}$ 7.29, $H^{\zeta3}$ 6.84, $N^{\varepsilon1}$ 132.4, C^{O} 176.1, C^{γ} 109.7, $C^{\delta2}$ 118.1, $C^{\varepsilon2}$ 141.9, $C^{\varepsilon3}$ 119.1, $F^{\varepsilon3}$ –124.2
^{5F}W	8.28	121.6	4.64	57.5	3.24, 3.21	29.7	$C^{\delta1}$ 129.3, $C^{\varepsilon3}$ 105.7, $C^{\eta2}$ 112.9, $C^{\zeta2}$ 115.5, $H^{\delta1}$ 7.31, $H^{\varepsilon1}$ 10.29, $H^{\varepsilon3}$ 7.31, $H^{\eta2}$ 7.02, $H^{\zeta2}$ 7.44, $N^{\varepsilon1}$ 129.8, C^{O} 176.1, C^{γ} 111.6, $C^{\delta2}$ 129.9, $C^{\varepsilon2}$ 135.5, $C^{\zeta3}$ 119.1, $F^{\zeta3}$ –124.7
^{6F}W	8.29	121.7	4.65	57.5	3.24, 3.24	29.6	$C^{\delta1}$ 127.8, $C^{\varepsilon3}$ 121.9, $C^{\zeta2}$ 100.6, $C^{\zeta3}$ 110.7, $H^{\delta1}$ 7.24, $H^{\varepsilon1}$ 10.26, $H^{\varepsilon3}$ 7.57, $H^{\zeta2}$ 7.21, $H^{\zeta3}$ 6.96, $N^{\varepsilon1}$ 130.7, C^{O} 176.1, C^{γ} 111.6, $C^{\delta2}$ 126.3, $C^{\varepsilon2}$ 138.8, $C^{\eta2}$ 119.1, $F^{\eta2}$ –121.5
^{7F}W	8.30	121.6	4.66	57.4	3.26, 3.26	29.7	$C^{\delta1}$ 128.3, $C^{\varepsilon3}$ 117.0, $C^{\eta2}$ 109.5, $C^{\zeta3}$ 122.6, $H^{\delta1}$ 7.30, $H^{\varepsilon1}$ 10.65, $H^{\varepsilon3}$ 7.42, $H^{\eta2}$ 6.99, $H^{\zeta3}$ 7.10, $N^{\varepsilon1}$ 124.1, C^{O} 176.1, C^{γ} 112.4, $C^{\delta2}$ 133.6, $C^{\varepsilon2}$ 127.1, $C^{\zeta2}$ 119.1, $F^{\zeta2}$ –133.9

Table S11. Observed cross-residue NOE cross-peaks with signal-to-noise ratios (S/N) in native peptide **1**, the ^{6F}Trp4-substituted variant **2c**, and the ^{4F}Trp9-substituted variant **3a**. Distances were estimated based on the S/N of the intra-residual NOE of Trp $H^{\zeta 2}$–$H^{\varepsilon 1}$, the distance was measured from the solution structure of Trpzip2 (PDB code 1LE1).[3] Distances in **2c** and **3a** that deviate by more than 0.3 Å from the native value are highlighted.

		Peptide **1**		Peptide **2c**		Peptide **3a**	
NOE between †		S/N	Estimated distance (Å)	S/N	Estimated distance (Å)	S/N	Estimated distance (Å)
Trp2 HD1	Trp11 HZ3	overlap	—	overlap	—	112	3.2
Trp2 HD1	Trp11 HE3	63	3.28	—	—	77	3.41
Trp2 HE1	Trp11 HZ3	61	3.29	123	3.33	83	3.36
Trp2 HE1	Trp11 HE3	29	3.73	55	3.80	40	3.80
Trp2 HH2	Trp11 HB3	31	3.69	90	3.50	60	3.55
Trp2 HZ3	Trp9 HB2	22	3.90	73	3.63	65	3.50
Trp2 HZ3	Thr10 HA	60	3.30	155	3.20	92	3.31
Trp2 HZ3	Trp11 H	49	3.42	126	3.31	92	3.31
Trp2 HZ3	Trp11 HB3	54	3.36	157	3.19	86	3.34
Trp2 HE3	Thr3 H	34	3.63	84	3.54	54	3.61
Trp2 HE3	Trp9 HB2	161	2.80	372	2.77	292	2.73
Trp2 HE3	Thr10 H	48	3.43	115	3.36	68	3.48
Trp2 HE3	Trp11 H	33	3.65	46	3.92	28	4.03
Trp2 HE3	Trp11 HA	50	3.40	artifacts	—	61	3.54
Trp4 HE1	Gly7 HA3	42	3.51	138	3.26	82	3.37
Trp4 HZ2	Gly7 HA3	96	3.05	205	3.05	169	2.99
Trp4 HH2	Gly7 HA3	32	3.67	substituted	—	38	3.83
Trp4 HH2	Lys8 HA	21	3.93	substituted	—	40	3.80
Trp4 HZ3	Lys8 HA	53	3.37	101	3.44	81	3.38
Trp4 HZ3	Trp9 H	38	3.56	88	3.52	65	3.50
Trp4 HZ3	Trp9 HD1	artifacts	—	110	3.39	95	3.29
Trp4 HZ3	Trp9 HE1	29	3.73	122	3.33	56	3.59
Trp9 HD1	Trp4 HZ3	101	3.03	218	3.02	141	3.08
Trp9 HE1	Trp4 HZ3	31	3.69	69	3.66	80	3.38
Trp9 HZ2	Trp2 HB2 *	37	3.58	25	4.34	7	5.08
Trp9 HZ2	Trp4 HB3 *	18	4.04	17	4.97	26	4.08
Trp9 HH2	Trp4 HB3	overlap	—	54	3.82	38	3.83
Trp9 HZ3	Trp2 HB3	54	3.36	91	3.50	43	3.75
Trp9 HZ3	Thr3 HA	83	3.13	190	3.09	71	3.45
Trp9 HZ3	Trp4 HB3	60	3.30	160	3.18	75	3.42
Trp9 HZ3	Trp4 H	55	3.35	132	3.29	63	3.52
Trp9 HE3	Trp2 HB2	167	2.78	316	2.84	substituted	—
Trp9 HE3	Thr3 H	35	3.61	69	3.66	substituted	—
Trp9 HE3	Trp4 HB3	42	3.51	66	3.69	substituted	—
Trp9 HE3	Trp4 H	24	3.85	132	3.29	substituted	—
Trp9 HE3	Thr10 H	31	3.69	86	3.53	substituted	—
Trp11 HH2	Ser1 HA	25	3.82	72	3.64	44	3.74
Trp11 HZ3	Ser1 HA	67	3.24	150	3.22	100	3.26
Trp11 HZ3	Trp2 H	26	3.80	68	3.67	34	3.90
Trp11 HZ3	Trp2 HE1	27	3.77	96	3.47	73	3.44
Thr3 H	Thr10 H	104	3.01	234	2.99	128	3.13
Thr3 H	Trp11 HA	15	4.16	41	3.99	20	4.26
Trp4 HA	Thr10 H	17	4.08	44	3.95	48	3.68
Glu5 H	Trp9 HA	25	3.82	53	3.83	47	3.70
Glu5 H	Trp9 H	88	3.10	diagonal	—	142	3.08
Reference NOEs							
Trp HZ2–HE1 (overall average)		131	2.90	280	2.90	202	2.90

† Resonances detected in F2 are shown on the left, in F1 on the right
* These NOEs could also be the result of spin diffusion

Table S12. Peptide library sequences with peptide masses M_{found} found in ESI-TOF analysis compared to calculated average molar masses M_{calcd}.

ID	Sequence	M_{calcd} (Da)	M_{found} (Da)
**1**	SWTWENGKWT WK–NH_2	1607.7919	1606.764
**2a**	SWT(^{4F}W)ENGKWT WK–NH_2	1625.7836	1625.756
**2b**	SWT(^{5F}W)ENGKWT WK–NH_2	1625.7836	1624.738
**2c**	SWT(^{6F}W)ENGKWT WK–NH_2	1625.7836	1624.730
**2d**	SWT(^{7F}W)ENGKWT WK–NH_2	1625.7836	1624.726
**3a**	SWTWENGK(^{4F}W)T WK–NH_2	1625.7836	1624.762
**3b**	SWTWENGK(^{5F}W)T WK–NH_2	1625.7836	1624.786
**3c**	SWTWENGK(^{6F}W)T WK–NH_2	1625.7836	1624.748
**3d**	SWTWENGK(^{7F}W)T WK–NH_2	1625.7836	1624.774
**4**	SWT(^{4F}W)ENGKWT (^{4F}W)K–NH_2	1643.7752	1643.756
**5**	S(^{4F}W)TWENGK(^{4F}W)T WK–NH_2	1643.7752	1642.758
**6a**	GG(^{4F}W)AGG	521.5070	521.204
**6b**	GG(^{5F}W)AGG	521.5070	521.206
**6c**	GG(^{6F}W)AGG	521.5070	521.213
**6d**	GG(^{7F}W)AGG	521.5070	521.204

3 Experimental Procedures

3.1 Materials

Fmoc-protected amino acids bearing orthogonal protection groups (Asn: Trt; Glu, Ser, Thr: *t*-Bu; Lys, Trp: Boc) were purchased from Carbolution Chemicals GmbH (Germany). Fmoc-4-fluoro-L-Trp, Fmoc-5-fluoro-L-Trp, Fmoc-6-fluoro-L-Trp, and Fmoc-7-fluoro-L-Trp were purchased from abcr GmbH (Germany). TentaGel® XV Rink amide resin was purchased from Iris Biotech GmbH (Germany). Fmoc-Gly-Wang polystyrene (LL) resin was purchased from CEM corp. (United States). All other solvents and reagents were purchased in HPLC grade quality from Carl Roth GmbH + Co. KG (Germany), Merck KGaA (Germany), and Fisher Scientific (United Kingdom). Solvents and reagents were used as received. Water was purified using a Milli-Q® Reference A+ system equipped with a Q-Gard® T2 cartridge (Merck KGaA, Germany). LC columns were purchased from Phenomenex Ltd. (Germany).

3.2 Software

Data were processed and visualized with Anaconda (Python 3.12). NMR data were processed with Bruker TopSpin 4.3 and analyzed using NMRFAM-Sparky 1.3 (Sparky 3.12).[4] ^{19}F NMR spectra were analyzed using MestReNova 14.2.

3.3 Solid-Phase Peptide Synthesis

TrpZip peptides were synthesized on TentaGel® XV Rink amide resin (0.05 mmol, 0.24 mmol/g) using a Liberty Blue™ automated microwave peptide synthesizer (CEM corp., United States). Microwave methods were adapted from Pham *et al.*[5]. Resin was swelled for at least 10 min in DMF prior to synthesis. Fmoc-protected amino acids were coupled as 0.2 M solutions in DMF for 3 min at 90 °C (Asn, Glu, Gly, Lys, Ser, Thr, Trp) or as 0.1 M solutions in DMF for 15 min at 75 °C (^{4F}Trp, ^{5F}Trp, ^{6F}Trp, ^{7F}Trp) using DIC (0.5 M) in DMF as activator, and Oxyma® (1.0 M) + DIPEA (0.1 M) in DMF as activator base. Fmoc protecting groups were removed using piperidine (20 vol%) in DMF for 2 min at 90 °C. Resin-loaded peptide was transferred into a 10-mL fritted syringe reactor, washed with DMF (3×5 mL), and DCM (3×5 mL), and dried under reduced pressure.

Random coil GGXAGG peptides were synthesized manually on preloaded Fmoc-Gly-Wang polystyrene (LL) resin (0.05 mmol, 0.3 mmol/g) in a 10-mL fritted syringe reactor. Resin was swelled for at least 10 min in DMF prior to synthesis. Ala, and Gly were coupled by treating the resin twice with a solution of Fmoc-protected amino acid (0.40 mmol, 8.0 equiv), Oxyma® (0.40 mmol, 8.0 equiv), and DIC (0.40 mmol, 8.0 equiv) in DMF (2.5 mL) for 1 h at room temperature. Fluorinated Trp derivatives were coupled by treating the resin with a solution of Fmoc-protected amino acid (0.13 mmol, 2.5 equiv), HOAt (0.13 mmol, 2.5 equiv), and DIC (0.13 mmol, 2.5 equiv) in DMF (2 mL) for 16 h. Fmoc protecting groups were removed by treating the resin three times with piperidine (20 vol%) in DMF (5 mL) for 10 min at room temperature. Coupling or deprotection was followed by washing the resin with DCM (3×5 mL), and DMF (3×5 mL). Resin-loaded peptide was washed with DMF (3×5 mL), and DCM (3×5 mL), and dried under reduced pressure

Dry resin-loaded peptide was cleaved and deprotected in TFA/TIS/Milli-Q water (95/2.5/2.5, v/v/v, 5 mL) for 3 h at room temperature, filtered, and washed with TFA (2×1 mL). Combined filtrates and washings were concentrated under nitrogen stream. The oily residue was dissolved in Milli-Q water + 0.1 vol% TFA and shaken for 2 h at room temperature to hydrolyze carbamate adducts from incomplete Trp(Boc) deprotection. Crude peptide solution was freeze dried under reduced pressure.

3.4 High-Performance Liquid Chromatography (HPLC)

Crude peptides were dissolved in Milli-Q water + 0.1 vol% TFA. The solution was filtered through a 0.2 µm PTFE membrane filter, and purified by reversed-phase HPLC using a Shimadzu chromatography system comprising a SCL-40 system controller, a SPD-40 UV/VIS detector, a LC-20AP preparative liquid chromatograph, a FCV-200AL prep quaternary valve, and a LH-40 liquid handler. Fractions were analyzed using a Hitachi Primaide chromatography system comprising a 1110 pump, a 1210 autosampler, and a 1430 diode array detector. A two-solvent system of Milli-Q water + 0.1 vol% TFA (solvent A), and acetonitrile + 0.1 vol% TFA (solvent B) was used as mobile phase in a linear gradient. HPLC gradients are listed in Table S13. Chromatograms were monitored at 220 nm using a deuterium (D2) lamp. TrpZip peptides were purified using

gradient **P1**, and analyzed using gradient **A1**. Random coil GGXAGG peptides were purified using gradient **P2**, and analyzed using gradient **A2**. Fractions containing pure peptide were pooled and freeze-dried under reduced pressure.

Table S13. HPLC columns and gradients used for peptide purification and analysis.

Name	Column	Flow (mL min^{-1})	Gradient
**P1**	Kinetex® 5 µm C18 100 Å 250×21.2 mm	20.0	20–38 %B over 18 min
**P2**	Kinetex® 5 µm C18 100 Å 250×21.2 mm	20.0	5 %B over 3 min, 5–45 %B over 14 min
**A1**	Kinetex® 5 µm C18 100 Å 250×4.60 mm	1.00	20–50 %B over 18 min
**A2**	Kinetex® 5 µm C18 100 Å 250×4.60 mm	1.00	5 %B over 3 min, 5–60 %B over 20 min

3.5 Mass Spectrometry

Peptide puritiy was determined by ESI-TOF mass spectrometry on an Agilent 6230 System with 1260 Infinity II LC system and 1100 DAD detector. Peptide samples were prepared as ∼1 mg/mL solutions in Milli-Q water + 0.1 vol% TFA.

3.6 UV Spectroscopy

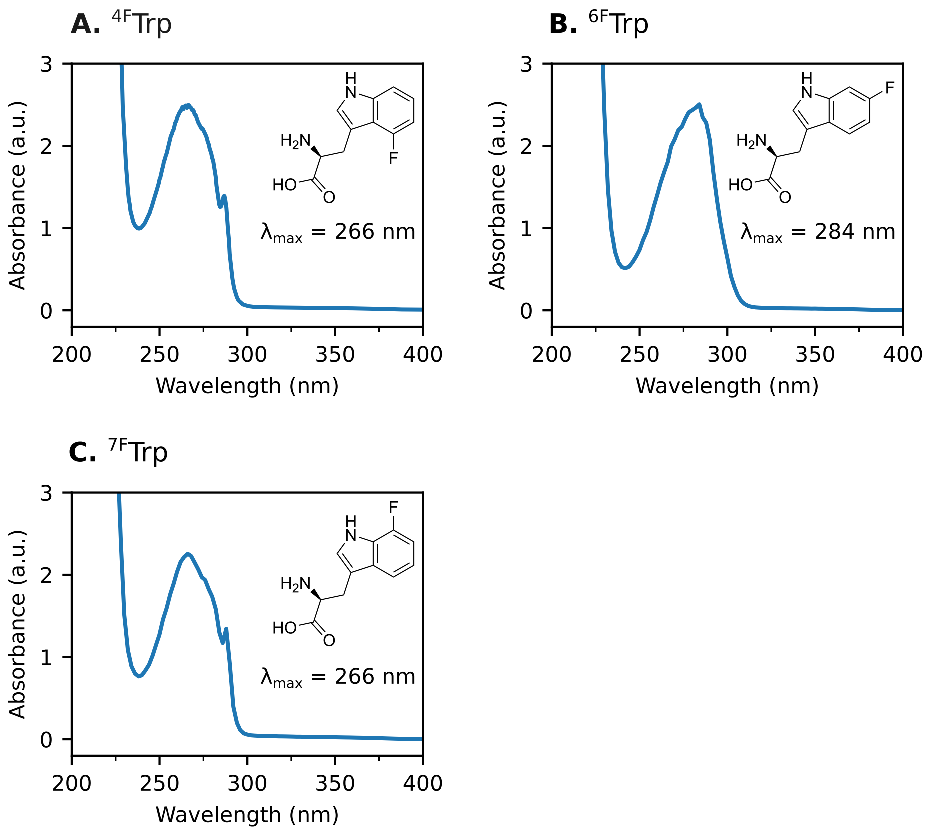

Figure S5. UV absorbance spectra of fluoro-Trp regioisomers (0.5 M) in solution in methanol. **A.** 4-fluoro-L-Trp. **B.** 6-fluoro-L-Trp. **C.** 7-fluoro-L-Trp.

Molar absorption coefficients $\varepsilon_{280\,\mathrm{nm}}$ of (fluorinated) Trp derivatives at 280 nm are listed in Table S14. Molar absorption coefficients of Trp and (5F)Trp have been reported elsewhere.[6,7] Molar absorption coefficients of (4F)Trp, (6F)Trp, and (7F)Trp were determined by measuring the absorbance of amino acid solutions in methanol with defined concentrations (0.10 mM, 0.25 mM and 0.50 mM) at 280 nm using an Eppendorf BioPhotometer plus with a disposable Eppendorf UVette® with 1.00 cm pathlength. Absorbance spectra

are shown in Figure S5. Molar absorption coefficients $\varepsilon_{280\,\mathrm{nm}}$ (L mol^{-1} cm^{-1}) were obtained by fitting experimental values to a linear model according to Lambert-Beer's law (1) with absorbance $A_{280\,\mathrm{nm}}$, amino acid concentration c (mol L^{-1}), and pathlength l (cm).

$$A_{280\,\mathrm{nm}} = c \cdot \varepsilon_{280\,\mathrm{nm}} \cdot l \tag{1}$$

Table S14. Molar absorption coefficients of fluorinated Trp derivatives.

Amino acid	$\varepsilon_{280\,\mathrm{nm}}$ (L mol^{-1} cm^{-1})
L-Trp	5500 [6]
4-Fluoro-L-Trp	(3518 ± 21)
5-Fluoro-L-Trp	(5530 ± 280) [7]
6-Fluoro-L-Trp	(4690 ± 68)
7-Fluoro-L-Trp	(2450 ± 220)

3.7 Peptide Stock Solutions

Peptide stock solutions were prepared by dissolving purified peptides (0.5 mg) in hexafluoro-2-propanol (500 µL). Stock concentrations were accurately determined by UV spectroscopy using an Eppendorf BioPhotometer plus monitoring the absorbance of Trp (and fluorinated derivatives thereof) at 280 nm.[6] Aliquots (10 µL) of stock solution were evaporated under nitrogen stream and redissolved in 20 mM phosphate buffer + 6 M GuHCl pH 7.0 (1.0 mL). The absorbance $A_{280\,\mathrm{nm}}$ of three aliquots was measured in a disposable Eppendorf UVette® (220 – 1600 nm) with 1.00 cm pathlength against 20 mM phosphate buffer containing 6 M GuHCl at pH 7.0 as a blank and averaged to calculate the stock concentration c (mol L^{-1}) using Lambert-Beer's law (2).

$$c = \frac{A_{280\,\mathrm{nm}}}{\varepsilon_{280\,\mathrm{nm}}} \cdot d \tag{2}$$

Molar absorption coefficients $\varepsilon_{280\,\mathrm{nm}}$ (L mol^{-1} cm^{-1}) were estimated as the sum of the molar absorption coeffients of (fluorinated) Trp residues listed in Table S14 for each peptide. Dilution factor $d = 100$.

3.8 Circular Dichroism (CD) Spectroscopy

CD experiments were performed on a Jasco J-810 spectropolarimeter, equipped with a Jasco PTC-423S Peltier thermostatted cell holder, and a Haake WKL 26 recirculating chiller, using a Hellma® 110-QS 1.00 mm quartz cuvette. CD samples were prepared by evaporating aliquots of peptide stock solutions under nitrogen stream and redissolving the residue in respective buffer. Samples were incubated for at least 12 h at 4 ℃ prior to CD experiments. Far-UV spectra of 60 µM peptide solutions in 20 mM phosphate buffer at pH 7.0 with, or without addition of 2 M GuHCl were acquired from 190 – 260 nm at 20 ℃. Final spectra were recorded as an average of four consecutive scans and baseline subtracted. Spectra were normalized relative to sample concentration c (mol L^{-1}), path length l (cm), and the number of backbone amide bonds n by converting the measured ellipticity θ (mdeg) to mean residue ellipticity $[\theta]$ (deg cm^2 dmol^{-1} res^{-1}) using Equation 3.

$$[\theta] = \frac{\theta}{c\,l\,n} \tag{3}$$

Variable-temperature measurements of 60 µM peptide solutions in 20 mM phosphate buffer at pH 7.0 containing 2 M GuHCl were conducted from 5 – 95 ℃ with a ramp speed of 1 ℃ min^{-1} by monitoring the ellipticity of the maximum at $\sim$227 nm. Data points were collected every 1 ℃. Variable temperature data was fit assuming a two-state transition from folded to unfolded state using the fit function f with temperature T (K), molar enthalpy ΔH (kcal mol^{-1}), melting temperature T_m (K), upper baseline u, lower baseline l, and the ideal gas constant R (1.9872 kcal K^{-1} mol^{-1}) according to Equations 4a–4c.[1]

$$k = \exp\left(\frac{\Delta H}{RT}\left(\frac{T}{T_\mathrm{m}} - 1\right)\right) \tag{4a}$$

$$y = \frac{k}{1+k} \tag{4b}$$

$$f(T, \Delta H, T_{\mathrm{m}}, u, l) = (u - l) \cdot y + l \tag{4c}$$

At least three independent variable-temperature measurements were carried out for each peptide, and the melting temperatures from the individual fits were averaged. The standard deviation of the mean was calculated according to Equation 5.

$$SD = \sqrt{\frac{1}{n-1}\sum_{i=1}^{n}(x_i - \bar{x})^2} \tag{5}$$

3.9 Fourier Transform Infrared (FTIR) Spectroscopy

FTIR spectra were recorded as described previously.[8] Peptides containing TFA from solid-phase synthesis were converted to their respective chloride salts by freeze-drying peptide solutions (1 mg mL^{-1}) in 7.5 mM HCl three times. Samples for FTIR measurements were prepared by dissolving peptides (1 mg) in D_2O (200 µL). A droplet was placed between two CaF_2 windows separated by a spacer of 100 µm thickness and inserted into a temperature-controlled stage (FTIRSP600, Linkam). Transmission FTIR spectroscopy was conducted on a Bruker Vertex 80V FTIR spectrometer at a spectral resolution of 2 cm^{-1}. An optical filter was used to restrict the spectral range in between 1950–0 cm^{-1}, 512 scans were recorded for both background and sample measurements and the scanner velocity was set to 80 kHz. Temperature-dependent FTIR recordings were carried out in the range between 5–95 °C with increments of 5 ($\pm$0.1) °C. After reaching the target temperature, a stabilization interval of 1 min was maintained for thermal equilibration before acquiring the spectrum of interest.

To account for temperature-dependent spectral changes of the solvent, spectra of pure D_2O were recorded at each temperature and subtracted from the sample peptide spectra in D_2O. Water vapor contributions were subtracted and a linear baseline correction was applied. Absorption difference spectra were obtained with the spectrum at 5 °C as a reference which was subtracted from the spectra at increasing temperatures. The resulting set of temperature-dependent spectra was subjected to singular value decomposition (SVD) by a MATLAB-based script.[8]

3.10 Nuclear Magnetic Resonance (NMR) Spectroscopy

Unless stated otherwise NMR spectra were recorded on a 700 MHz AVANCE III spectrometer (Bruker BioSpin) equipped with a TCI ($^1H/^{13}C/^{15}N$) cryo probe at 300 K. Peptides were dissolved in 10 % D_2O/90 % H_2O typically with a concentration of 2 mM and measured in 5 mm NMR tubes (abcr GmbH). Proton spectra were recorded with water suppression using a double Watergate 3-9-15, 32 transients, 8192 complex points, a spectral width of 16.63 ppm and a recycle delay of 1.3 s. One-dimensional ^{13}C spectra were recorded using the standard Bruker pulse sequence zgpg30, 32768 complex points, a spectral width of 304.2 ppm, a recycle delay of 1 s and 14 000 to 80 000 transients. Standard 2D NMR spectra were recorded: ^1H-^1H TOCSY spectra with Watergate 3-9-15 using a mixing time of 120 ms, 1024 × 256 complex points, spectral widths of 16.63 ppm, 4 scans and a recycle delay of 1.1 s; an identical experiment with a mixing time of 12 ms served as ^1H-^1H COSY; a ^1H-^1H NOESY with Watergate 3-9-15 using a mixing time of 120 ms, 2048 × 250 complex points, spectral widths of 16.63 ppm and 11.9 ppm, 96 scans and a recycle delay of 1.3 s; a ^1H-^{13}C HSQC optimized for aliphatic side chains with 512 × 110 complex points, spectral widths of 15.94 ppm and 61.73 ppm, 32 scans, a recycle delay of 1.1 s and a ^{13}C offset of 15 ppm; a ^1H-^{13}C HSQC optimized for aromatic side chains width 512 × 50 complex points, spectral widths of 15.94 ppm and 31.55 ppm, 32 scans, a recycle delay of 1.1 s and a ^{13}C offset of 116 ppm; a ^1H-^{15}N HSQC with 512 × 64 complex points, spectral widths of 16.63 ppm and 49.62 ppm, 16 scans, a recycle delay of 1.1 s and a ^{15}N offset of 118.4 ppm. Random coil chemical shifts were measured at 298 K for consistency with previously reported values.[2]

^{19}F spectra were recorded on a JEOL ECZ600 spectrometer equipped with a SUPERCOOL 1H/BB probe using a spectral width of 402.4 ppm, 128 transients and a recycle delay of 7.5 s.

Spectra were referenced to 2,2-dimethyl-2-silapentane-5-sulfonic acid (DSS using an external sample of 0.5 mM DSS and 2 mM sucrose in H_2O/D_2O. Indirect chemical shift referencing was applied to ^{13}C and ^{15}N using the IUPAC-IUB recommended chemical shift referencing ratios Ξ of 0.251 449 530 (^{13}C) and 0.101 329 18 (^{15}N), respectively.[9] The chemical shift assignments are made publicly available at the BioMagResBank[10] under the accession codes 52965 (**2a**), 52989 (**2b**), 52988 (**2c**), 52987 (**2d**), 52986 (**3a**), 52992 (**3b**), 52991 (**3c**), 52990 (**3d**), 52994 (**6a**), 52997 (**6b**), 52996 (**6c**), 52995 (**6d**).

4 Abbreviations

^{4F}Trp, ^{4F}W	4-Fluoro-L-tryptophan
^{5F}Trp, ^{5F}W	5-Fluoro-L-tryptophan
^{6F}Trp, ^{6F}W	6-Fluoro-L-tryptophan
^{7F}Trp, ^{7F}W	7-Fluoro-L-tryptophan
Boc	*tert*-Butyloxycarbonyl
CD	Circular dichroism
CSD	Chemical shift deviation
DCM	Dichloromethane
DIC	*N*,*N'*-Diisopropylcarbodiimide
DIPEA	*N*,*N*-Diisopropylethylamine
DMF	*N*,*N*-Dimethylformamide
DSS	2,2-dimethyl-2-silapentane-5-sulfonic acid
ESI-TOF	Electrospray ionization time-of-flight
Fmoc	Fluorenylmethyloxycarbonyl
GuHCl	Guanidine hydrochloride
HPLC	High-performance liquid chromatography
t-Bu	*tert*-Butyl
NMR	Nuclear magnetic resonance
NOE	Nuclear Overhauser effect
PTFE	Polytetrafluoroethylene
TFA	Trifluoroacetic acid
TIS	Triisopropyl silane
Trt	Trityl

Canonical amino acids were abbreviated using the IUPAC-IUB recommended one-letter or three-letter codes.

References

[1] N. J. Greenfield, *Nat. Protoc.* **2006**, *1*, 2527–2535.

[2] D. S. Wishart, C. G. Bigam, A. Holm, R. S. Hodges, B. D. Sykes, *J. Biomol. NMR* **1995**, *5*, 67–81.

[3] A. G. Cochran, N. J. Skelton, M. A. Starovasnik, *Proc. Natl. Acad. Sci. USA* **2001**, *98*, 5578–5583.

[4] W. Lee, M. Tonelli, J. L. Markley, *Bioinformatics* **2015**, *31*, 1325–1327.

[5] T. L. Pham, S. Fazliev, P. Baur, P. Comba, F. Thomas, *ChemBioChem* **2023**, *24*, e202200588.

[6] C. N. Pace, F. Vajdos, L. Fee, G. Grimsley, T. Gray, *Protein Sci.* **1995**, *4*, 2411–2423.

[7] A. D. Richaud, S. Mandal, A. Das, S. P. Roche, *ACS Chem. Biol.* **2023**, *18*, 2555–2563.

[8] L. Schubert, J.-L. Chen, T. Fritz, F. Marxer, P. Langner, K. Hoffmann, A. P. Gamiz-Hernandez, V. R. I. Kaila, R. Schlesinger, J. Heberle, *J. Phys. Chem. B* **2023**, *127*, 8358–8369.

[9] J. L. Markley, A. Bax, Y. Arata, C. W. Hilbers, R. Kaptein, B. D. Sykes, P. E. Wright, K. Wüthrich, *Pure Appl. Chem.* **1998**, *70*, 117–142.

[10] J. C. Hoch, K. Baskaran, H. Burr, J. Chin, H. Eghbalnia, T. Fujiwara, M. Gryk, T. Iwata, C. Kojima, G. Kurisu, D. Maziuk, Y. Miyanoiri, J. Wedell, C. Wilburn, H. Yao, M. Yokochi, *Nucleic Acids Res.* **2023**, *51*, D368–D376.
